# Impairment of Intermediate Filament Expression Reveals Impact on Cell Functions Independent from Keratinocyte Transformation

**DOI:** 10.3390/cells13231960

**Published:** 2024-11-26

**Authors:** Charlotte Klein, Imke Ramminger, Shuoqiu Bai, Thorsten Steinberg, Pascal Tomakidi

**Affiliations:** 1 Division of Oral Biotechnology, Center for Dental Medicine, Medical Center—University of Freiburg, Faculty of Medicine, University of Freiburg, Hugstetterstr. 55, 79106 Freiburg, Germany; charlotte.klein@uniklinik-freiburg.de (C.K.); imke.ramminger@uniklinik-freiburg.de (I.R.); shuoqiu.bai@uniklinik-freiburg.de (S.B.); pascal.tomakidi@uniklinik-freiburg.de (P.T.); 2 Department of Operative Dentistry and Periodontology, Center for Dental Medicine, Medical Center, Faculty of Medicine, University of Freiburg, Hugstetter Straße 55, 79106 Freiburg, Germany; 3 Faculty of Biology, University of Freiburg, Schaenzlestr. 1, 79104 Freiburg, Germany

**Keywords:** intermediate filaments, keratinocytes, transformation, keratin, vimentin, involucrin, proliferation, differentiation, chromatin, apoptosis

## Abstract

Although cytoplasmic intermediate filaments (cIFs) are essential for cell physiology, the molecular and cell functional consequences of cIF disturbances are poorly understood. Identifying defaults in cell function-controlled tissue homeostasis and understanding the interrelationship between specific cIFs and distinct cell functions remain key challenges. Using an RNAi-based mechanistic approach, we connected the impairment of cell-inherent cIFs with molecular and cell functional consequences, such as proliferation and differentiation. To investigate cIF disruption consequences in the oral epithelium, different cell transformation stages, originating from alcohol-treated oral gingival keratinocytes, were used. We found that impairment of keratin (KRT) KRT5, KRT14 and vimentin (VIM) affects proliferation and differentiation, and modulates the chromatin status. Furthermore, cIF impairment reduces the expression of nuclear integrity participant lamin B1 and the terminal keratinocyte differentiation marker involucrin (IVL). Conversely, impairment of IVL reduces cIF expression levels, functionally suggesting a regulatory interaction between cIFs and IVL. The findings demonstrate that the impairment of cIFs leads to imbalances in proliferation and differentiation, both of which are essential for tissue homeostasis. Thus, targeted impairment of cIFs appears promising to investigate the functional role of cIFs on cell-dependent tissue physiology at the molecular level and identifies putative interactions of cIFs with epithelial differentiation.

## 1. Introduction

Based on the “*Global Burden of Disease Study*” from 2016, it has been proven that alcohol is one of the leading risk factors in terms of mortality or massive impairment of life. In the context of mortality, the study was also able to show that cancer, including lip and oral cavity cancer, was one of the causes of death in populations aged 50 and over within the 195 countries and regions included in the study [1]. Interestingly, for East Asian countries, detailed analyses have shown that there is a genotypic predisposition in which a certain alcohol dehydrogenase (ALDH) genotype, namely ALDH2-rs-671, is associated with an increased risk of head and neck, esophageal and lung cancer in men who frequently consumed alcohol [2]. The causalities just described with regard to the life-threatening effects of alcohol illustrate the clinical-scientific need for action to deal intensively with the molecular basis of oral carcinogenesis and to gain new insights.

Usually, manifest oral carcinomas or cell lines derived from this tissue represent the final stage of tumor progression, regardless of their potential for metastasis. Hence, there are often no suitable examination systems which allow analyses of molecular and cell functional changes in the earliest possible stages, i.e., at the beginning of oral carcinogenesis [3]. Against this background, we developed a cell system in 2003 which is based on immortalized, i.e., permanently cultivatable but non-tumorigenic keratinocytes, of the oral mucosa (GKs) [4]. Three stable phenotypes, which differ in terms of their morphology and their molecular composition, could be discriminated by alcohol treatment. These three phenotypes are devoid of a neoplastic potential, but represent different stages of tumor progression due to their molecular characteristics. In detail, the three phenotypes are the parental cell line, i.e., gingival keratinocytes (GKs), which have all the molecular characteristics of the native tissue [5]. In addition, the fibroblastoid (fibroblast-like) phenotype (FIB) shows clear molecular changes compared to GKs, and the epitheloid (epithelium-like) phenotype (EPI) represents an intermediate of GKs and FIBs. The predominant molecular differences between FIBs and GKs as well as EPIs among others comprise a strong loss in E-cadherin expression in conjunction with a significant up-regulation of epithelial-atypical cIFs, such as VIM. Therefore, in comparison with EPIs, the FIB phenotype represents an even more advanced stage in cell transformation, the process which includes all genetic and molecular changes from the normal to the malignant tumor cell [3,6]. This finding was supported by a more extensive molecular biological analysis of the relevant EMT-associated transcription factors SNAIL1, ZEB1 and TWIST1 [7], which were also significantly up-regulated in FIB cells compared to EPI and GK cells. As further molecular distinction, a strongly reduced expression of the cIFs KRT5 and KRT14, which are typical for the stratified gingival epithelium [8], was identified in FIBs [5,9,10]. The gain of atypical and the reduction or loss of typical biomarkers of squamous epithelia, such as cIFs, characterize squamous epithelial tissues like the epidermis of skin but also oral mucosa during epithelial to mesenchymal transition (EMT) [11]. The same situation fairly applies to malignant transformation, which ends up in squamous cell carcinoma (SCC) [12] or oral squamous cell carcinoma (OSCC), respectively [13].

In eukaryotic cells, the cytoskeleton comprises three types of polymer-based filaments, (i) actin microfilaments, (ii) tubulin microtubules and (iii) intermediate filaments (IFs), which all together control their mechanical properties and thus the cell shape [14,15]. There is a growing body of evidence that cytoskeletal integrity governs cellular functions like proliferation, migration and energy production through mitochondria [16]. Therefore, it seems plausible that the loss of cytoskeletal integrity is associated with diseases like neurodegenerative diseases such as Alzheimer’s disease [17]. In the cytoskeleton, and here in particular actin, recent research shows that it is involved in the regulation of mitochondrial function, with mitochondrial dysfunction being one of the many facets of aging [18].

CIFs, which are larger than actin and smaller in size than tubulin regulate cell shape, and due to their mechanosensitive properties are also indispensable for cell mechanics and integrity [19,20]. The control of the cell and tissue mechanical properties of cIFs arises from their elasticity and the ability to interact with each other, thereby forming a viscoelastic intracellular meshwork. Since the length of IFs scales from the nanometer to micrometer, they are much more flexible than actin and microtubules, and unlike microfilaments and microtubules, they polymerize through self-assembly [21], i.e., they do not require ATP or GTP. Generally, IFs comprise six cell-specific types, among others, orphans (type 6), nuclear lamins (type 5, nuclear [n] IFs), neurofilaments (type 4), VIM (type 3), which is found in mesenchymal cells (e.g., connective tissue fibroblasts), and the basic (type 2) and acidic (type 1) keratins, which are found in epithelial cells (e.g., skin/epidermal or gingival epithelial keratinocytes). In *homo sapiens*, type 2 and type 1 keratins are encoded by a total of 54 keratin genes, whereas keratins form heterodimers comprising class 1 and 2 filaments [22].

In addition to their above-mentioned function as a mechanical stabilizer of cell shape and integrity, cIFs also have signal-transducing functions. This is due to the fact that cIFs have been found to be connected to adherens junctions and desmosomes, which reveals that they are involved in the mechanosensing and mechanotransducing activities emerging from cell-to-cell contacts [23]. Regarding the involvement of cIFs in cellular mechanosignaling, it could be shown in connection with desmosomes and here a desmoplakin (DP)-mutant that the disruption of the DP-cIF connection in keratinocyte monolayers compared to the control leads to a loss of keratinocyte stratification and to an induction of early and terminal keratinocyte differentiation. These processes were mediated with the involvement of serum response factor (SRF) signaling, as shown by SRF inhibition studies [24].

In addition, it could be shown that keratin filaments cooperate with actin filaments in cell migration and that the keratin filament system forms an elastic cage around the cell nucleus, which buffers the contractile forces of the more rigid actomyosin system [25]. For cell migration, integrin-driven interaction between the cell and extracellular matrix (ECM) is essential. Here, knockdown of the vesicle-trafficking protein GGA3 (Golgi-localized gamma-ear containing Arf-binding protein 3) was able to show that in addition to alpha (α) integrin subunits, such as α2 and α5, the β1 integrin in particular plays a major role [26]. If mutations occur in the cyto-protective keratins, this results in cell dysfunctions, the so-called keratinopathies, of which more than 60 are known to date. Mutations are found in the case of suprabasal early epithelial differentiation markers KRT1 and KRT10, but also in two basal cell-specific keratins KRT5 and KRT14 either at the beginning or at the end of the rod domain. Such mutations cause skin diseases, whereas mutations in the KRT5 gene are also considered a risk factor for the development of basal cell carcinoma [27,28], and mutations in both KRT5 and KRT14 are frequently associated with epidermolysis bullosa simplex [29].

With regard to the type 3 cIF VIM, the previous scientific opinion that it was a nonessential protein has changed fundamentally. This is because research over the last three decades has shown that VIM plays a key role in mechanotransduction and migration. Ostrowska-Podhorodecka et al., and Ridge et al. have extensively reviewed these issues [30,31]. For example, VIM coordinates the proliferation of fibroblasts and the differentiation of keratinocytes in wound healing [32], and is involved in the regulation of integrin-based focal adhesions (FAs). Regarding FAs, VIM controls cell adhesion mediated through the fibronectin receptor α5β1 by binding to the integrin through its serine residue 38 [33].

nIFs, the lamins, are of major importance for nuclear integrity, since their depletion strikingly alters nuclear morphology and causes ruptures and blebs of the nuclear envelope [34]. Further, it could be shown that, in addition to chromatin instability, lamin B1 (LMNB1) defects or silencing cause a delay in the S phase and thus have a negative impact on cell proliferation [35,36]. Moreover, LMNB1 depletion increases apoptosis [37], whereas apoptosis increases could be observed in response to the induction of DNA double strand breaks [35]. Recent work showed that the transcriptional coactivator yes-associated protein (YAP) and the *transcriptional* coactivator with PDZ-binding motif (TAZ) regulate LMNB1 transcription in conjunction with ARP2/3 complex component ACTR2 (involved in actin filament nucleation). Actin-related protein 2 (ACTR2) is involved in building the peri-nuclear actin cap, important for nuclear deformation protection [38].

While basal cell-specific KRT5 and KRT14 are expressed within the less differentiated proliferative basal region of squamous epithelia, including oral mucosal gingival epithelium [39], biomarkers, such as loricrin (LOR) and IVL, are indicators of progressive late, i.e., terminal differentiation. Similar to IVL, LOR is a component of the cornified envelope (CE) in squamous epithelia, such as the epidermis of the skin or the keratinized gingiva of the oral mucosa [40,41]. The CE is a macromolecular protein–lipid complex that provides mechanical resistance to the squamous epithelial *stratum granulosum*. Studies on mouse models reveled that LOR defaults are associated with delayed acquisition of the epithelial barrier function. This feature becomes also evident in case of IVL dysfunction in response to down-regulation of expression in patients suffering from atopic dermatitis (AD) [42]. Moreover, LOR mutations have been found in *keratoderma hereditarium mutilans* with ichthyosis (Vohwinkel’s disease), a genodermatosis, characterized inter alia by hyperkeratosis of the palms and soles [43].

Although knowledge of the cellular function of cIFs has increased enormously in recent years, very little is known about cIF impairment in cells that represent different stages of transformation. Therefore, we have investigated here the molecular and cell functional consequences of the intracellular down-regulation of the epithelial-specific KRT5 and KRT14 as well as the mesenchymal cIF VIM and IVL in alcohol-treated squamous epithelial keratinocytes.

This is because other studies have shown that siRNA-mediated knockdown of numerous key molecules has shown that significant down-regulation has a decisive influence on cancer progression [44,45,46,47].

## 2. Materials and Methods

### 2.1. Cell Culture

The parental human oral gingival keratinocyte cell line was immortalized with the E6/E7 gene of the human papilloma virus type 16 (ihGK) [4], hereinafter referred to as GKs. GK derivates were established by chronical ethanol treatment resulting in an epithelium-like phenotype (EPI) and a fibroblast-like phenotype (FIB), as previously described [5]. GKs were cultivated in passages 42–56 in Keratinocyte Growth Medium 2 (KGM-2, PromoCell, Heidelberg, Germany, #C-20211) supplemented with SupplementMix (PromoCell, Heidelberg, Germany, #C-39016), 0.06 mM CaCl2 (PromoCell, #C-34005) and 100 µg mL^−1^ kanamycin (Sigma-Aldrich, St. Louis, MO, USA, #K0254). FIBs were used in passages 177–189 and EPIs in passages 155–165, and cultivated in Dulbecco’s modified Eagle medium (DMEM, Gibco, Waltham, MA USA, #22320-022) supplemented with 1x GlutaMax (Gibco, #35050-038), 10% fetal calve serum (Bio&Sell, Feucht, Germany, #BS.FCS 5.500 EUA) and 100 µg mL^−1^ kanamycin. All cells were cultured under standard cell culture conditions of 5% CO_2_ at 37 °C.

### 2.2. siRNA Treatment

For siRNA experiments, cells were seeded in 6-well culture plates at 2.1 × 10^4^ cells/cm^2^ for GKs and FIBs or 2.6 × 10^4^ cells/cm^2^ for EPIs and incubated overnight. After 24 h of growth, siRNA transfection was performed with a final concentration of either 100 nM (ON-TARGET plus SMART pool Human VIM, Dharmacon (Lafayette, CO, USA), #L-003551-00-0005) or 200 nM siRNA (Silencer Select siRNAs, Life Technologies GmbH, Darmstadt, Germany, #4392420, siRNA KRT5 ID: S7956 and S7958, siRNA KRT14 ID: S7985, siRNA IVL ID: S7940, siRNA negative control #4390844) according to the manufacturer’s protocols. For transfection, Lipofectamine RNAiMax (Life Technologies GmbH, Darmstadt, Germany, #13778-150) and Opti-MEM (Life Technologies GmbH, Darmstadt, Germany, #31985-047) were used in kanamycin-free medium. After 72 h, siRNA-treated cells were lysed for RNA or protein isolation or fixed for immune fluorescence staining. In the siRNA validation for the individual markers, the commercial siRNAs were selected in such a way that an inhibition efficiency of at least 70% was achieved in all cell types under study. This significant gene knockout was achieved for the analyzed markers KRT5, KRT14 and IVL with more than 80% inhibition; only VIM in the FIB cells could only be inhibited by 50%. This is probably due to the extremely high baseline expression of this intermediate filament in this cell type. However, even a 50% inhibition of a central IF can have significant effects on associated signaling pathways, which could also be shown in this study. The efficiency levels have been analyzed by Western blot and evaluated by densitometry. The data can be retraced in Supplemental Figure S3.

### 2.3. RNA Isolation and qPCR

mRNA was isolated with the RNeasy Plus Mini Kit (Qiagen, Hilden, Germany, #74034) according to the manufacturer’s protocol. QIAshredder tubes (Qiagen, Hilden, Germany, #79656) were used in advance to get rid of remaining cell fragments. RNA was diluted in 30–40 µL RNase-free water (Qiagen, Hilden, Germany, #129114). Concentration measurement was accomplished with the QiaExpert system (Qiagen, Hilden, Germany). The integrity of the isolated mRNA was verified with the 4150 TapeStation system (Aqilent, Santa Clara, CA, USA) according to the manufacturer’s protocol before cDNA Synthesis. Total RNA of 1 µg was synthesized into cDNA with the Revert Aid First Strand cDNA Synthesis Kit (Thermo Scientific, Darmstadt, Germany, #K1622). qPCR was performed in technical doublets of each reaction with 10 ng cDNA per well and an SYBR Green (Qiagen, Hilden, Germany, #1065806) reaction cocktail. All primers were purchased from Qiagen, Hilden, Germany (RT2 qPCR Primer Assay for Human: ACTR2 #PPH02650A, CDH1 #PPH00135F, ITGB1 # PPH00650B, ITGH3 #PPH00178D, IVL #PPH01911A, KRT1 #PPH06951F, KRT10 #PPH05868E, KRT14 #PPH02389A, KRT5 #PPH02625F, LMNB1 #PPH00278B, LOR #PPH06894F, VIM #PPH0417F, YAP1 #PPH13459A, SNAIL1 #PPH02459B, ZEB1 #PPH01922A, TWIST1 #PPH02132A, ACTB #PPH00073G, RPL13A #PPH01020B, TBCB #PPH08908A). For analysis, the relative expression levels of each gene and condition were normalized to the housekeeping genes (ACTB, RPL13A, TBCB) and calculated as ∆Ct values. The ∆Ct values were then used to analyze the ratio between the different conditions and the negative control (nt siRNA) and were calculated as ∆∆CT values.

### 2.4. Protein Isolation and Western Blot

Whole cell protein was extracted with RIPA buffer (Sigma-Aldrich, #R0278L) supplemented with phosphatase (PhosSTOP™, Sigma-Aldrich, #04906845001) and protease (cOmlete™ Mini Protease inhibitor-cocktail, Sigma-Aldrich, Sigma-Aldrich, St. Louis, MO, USA, #04693124001) inhibitors. Cells were lysed for 10 min on ice and scraped off the well plates, followed by 10 min of centrifugation at 10.000 rpm. Nuclear protein extraction was accomplished with the NE-PER Nuclear and Cytoplasmic Extraction Kit (Thermo Scientific, Darmstadt, Germany, #78833), according to the manufacturer’s protocol, supplemented with a Halt Protease and Phosphatase Inhibitor Cocktail (Thermo Scientific, Darmstadt, Germany, #1861281). Concentration measurement was performed with the Pierce™ BCA Protein Assay Kit (Thermo Scientific, Darmstadt, Germany, #23227) and the Pre-Diluted Protein Assay Standards: Bovine Serum Albumin (BSA) Set (Thermo Scientific, Darmstadt, Germany, #23208). Concentration was measured at 562 nm excitation in 96-well plates with an Infinite M200 plate reader (Tecan, Männerdorf, Switzerland, #30016056). Then, 10 µg whole cell protein extract or nuclear protein extract was mixed with 4xLaemmli (Bio-Rad, Hercules, CA, USA, #1610747), supplemented with 1:20 DTT (Sigma-Aldrich, #43815-1G) and denatured at 95 °C for 5 min. Proteins were loaded on a 4–15% Criterion™ TGX Stain-free™ Protein Gel (Bio-Rad, Munich, Germany, #5678084) and electrophoresis was done at 90 V for 20 min, followed by 180 V for 40 min with the Bio-Rad Criterion™ cell system. Proteins were transferred on a 0.45 µm PVDF membrane (Bio-Rad, Munich, Germany, #1704275) with the Bio-Rad turbo station and transferred with the pre-programmed Midi-gel protocol. Afterwards, the membrane was rinsed with sterile water for 10 min and total protein was detected with the ChemiDoc™ Touch imaging system (Bio-Rad, Munich, Germany) before a 2 h incubation step at RT with 5% Bovine Serum albumin (Sigma-Aldrich, St. Louis, MO, USA, #A7906) in TBS buffer (Bio-Rad, Munich, Germany, #1706435), supplemented with 0.05% Tween-20 (TBST, Bio-Rad, Munich, Germany, #1706435). Afterwards, primary antibody incubation was carried out overnight in 0.5% BSA in TBST buffer at 4 °C. Different primary antibodies were used at different concentrations (anti-Vim, Abcam (Cambridge, UK) #ab92547 1:3000; anti-Keratin 1, Abcam ab93652 1:1000; anti-Keratin 10, Abcam #ab76318 1: 2000; anti-Keratin 5, Abcam #ab52635 1:5000; anti-Keratin 14, Invitrogen MA5-11599 1:5000; anti-Involucrin, (Abcam Cambridge, UK) ab181980 1:5000; anti-Loricirn, Abcam ab137533 1:1000; anti-E-Cadherin, Abcam ab76055 1:5000; anti-Integrin ß1, Abcam ab179471 1:2000; anti-YAP (G-6), Santa Cruz (Santa Cruz, CA, USA) sc376830 1:500; anti-Lamin B1, Abcam ab133741 1:5000; anti-ß-Tubulin, Abcam ab6046 1:10.000). After three TBST washes, secondary antibody incubation with WesternSure^®^ HRP goat-anti-rabbit IgG (Li-Cor, #926-80011) or WesternSure^®^ HRP goat-anti-mouse IgG (Li-Cor, #926-80010) antibodies at 1:5000 was carried out for 1 h. Protein detection was conducted after two TBST washes and one TBS washing step with Clarity Western ECL Substrate, Peroxide Reagent and Luminol/Enhancer Reagent (Bio-Rad, Munich, Germany, #1705061) at 1:1 ratio for 4 min in a dark chamber. Proteins of interest were detected with the ChemiDoc™ Touch imaging system. Analysis was performed with the Image Lab analysis Software V3.01 (Bio-Rad, Munich, Germany) and the protein of interest amount was normalized to total protein amount of the according protein lane and compared to negative control in the experiment.

### 2.5. Indirect Immunofluorescence

GK, EPI and FIB cells were cultivated in 6-well plates on sterile coverslips. After siRNA transfection and 72 h growth, cells were washed three times with pre-warmed PBS and fixed with either 4% paraformaldehyde (PFA) (Carl Roth, Karlsruhe, Germany, #0335.1) in PBS for 20 min at RT or ice cold 100% Methanol (VWR, Radnor, PA, USA, #67-56-1) for 10 min at RT. After three rinses with PBS, cells were permeabilized with either 0.1% Triton-X-100 (Sigma-Aldrich, St. Louis, MO, USA, #9002-93-1) in PBS for 15 min at RT or 0.1% Triton-X-100 with 5% BSA (Sigma-Aldrich, #A8412) in PBS for 30 min at RT. Cells treated with 0.1% Triton-X-100 in PBS only were washed three times with PBS and incubated with 2% BSA for 1 h at RT. Afterwards, cells were directly incubated with the primary antibody in 0.5% BSA in PBS overnight at 4 °C. Cells treated with 0.1% Triton-X-100/ 5% BSA in PBS were washed three times with PBS and incubated with the primary antibody in 2% BSA in PBS overnight at 4 °C. Primary antibodies were used at different concentrations (PFA fix: anti-Vim, Abcam, Cambridge, UK, #ab92547 1:500; anti-Keratin 1, Abcam #ab93652 1:200; anti-Keratin 10, Abcam #ab76318 1: 200; anti-Keratin 5, Abcam #ab52635 1:250; anti-Keratin 14, Invitrogen MA5-11599 1:250; anti-Involucrin, Abcam ab181980 1:200; anti-Loricirn, Abcam ab137533 1:250; anti-E-Cadherin, Abcam ab76055 1:100; anti-Integrin ß1, Abcam ab179471 1:50; MeOH fix: anti-YAP (G-6), Santa Cruz sc376830 1:100; anti-Lamin B1, Abcam ab133741 1:150, Abcam ab32042 1:250; anti-cleaved caspase 3 antibody). The next day, the cells were washed three times with PBS and incubated with the secondary antibody goat-anti-mouse 488 (invitrogen, #A11029) or goat-anti-rabbit 488 (Invitrogen, #A11008) and phalloidin-iFluor 594 (abcam, #ab176757) in either 0,5% BSA in PBS or 2% BSA in PBS (according to primary antibody) for 1 h at RT in the dark. Afterwards, cells were washed three times with PBS and incubated with a 300 mM DAPI (Thermo Fischer Scientific, Darmstadt, Germany, #D3571) in PBS solution for 15 min at RT in the dark. Next, the cells were washed twice with PBS and once with distilled water and embedded on glass slides with Fluoromount-G^®^ (SouthernBiotech, Birmingham, AL, USA, #0100-01). Microscopy was performed with the Keyence BZ-9000 fluorescence microscope (KEYENCE GmbH, Neu-Isenburg, Germany) at different magnifications. Equal exposure times for the protein of interest were used in biological replicates, while exposure times for nuclei and actin were varied between different conditions. For the analysis of nuclear pyknosis, the exposure time for the nuclei was the same in all biological replicates.

### 2.6. Proliferation Assays

Proliferation analysis was performed with Alamar blue (Bio-Rad, Munich, Germany, #BUF012B) and the Quant-it PiccoGreen dsDNA Assay Kit (Thermo Fischer Scientific, Darmstadt, Germany, #P7589). Alamar blue is a resazurin-based reagent, which enters live cells. Resazurin is reduced to the highly fluorescent form resofurin by life cells. The fluorescence intensity was measured with a plate reader with an exciting wavelength of 560 nm and emission wavelength of 590 nm. GKs and FIB were seeded at 2.1 × 10^4^ cells/cm^2^ and EPIs at 2.6 × 10^4^ cells/cm^2^ in a 12-well plate. After 72 h of siRNA transfection, 10% Alamar blue reagent was added to complete medium of the according cell derivate. Cells were incubated for 2.5 h and the fluorescence intensity of the medium was measured with an Infinite M200 plate reader (Tecan, Männderdorf, Switzerland, #30016056). The cells were washed three times with pre-warmed DPBS and the PiccoGreen Assay was conducted afterwards according to the manufacturer’s protocol. With the PiccoGreen Assay, the whole DNA amount of a sample can be measured by fluorescence intensities at an Ex/Em of 480/520 nm. Fluorescence intensities were measured with an Infinite M200 plate reader.

### 2.7. Histone Extraction and Quantification

After 72 h of siRNA transfection, histones were extracted with a Histone Extraction Kit (Abcam, Cambridge, UK, #ab113476) according to the manufacturer’s protocol. The protein amount was measured with the Pierce™ BCA Protein Assay Kit as previously described. Different histone modifications were analyzed with fluorometric Quantification Kits from Abcam Cambridge, UK, according to the manufacturer’s protocol (H3K4me3 #ab115057, H3K9me3 #115065, H3K9ac #115105, H3K27me3 #115073). For normalization, the Histone H3 Total Quantification Kit (Abcam, Cambridge, UK, #ab115092) was used. For all kits, 0.25 µg Histone extract was used in technical duplicates.

### 2.8. Statistical Analysis

All experiments were performed with a minimum of three biological replicates. For statistical analysis, Micrsosoft Excel 2016 and Graph Pad Prism version 7.05 were used. A normality Shapiro–Wilk test was used, and to test between the different conditions, an unpaired *t*-test was used.

## 3. Results

### 3.1. Cell Morphology as Discriminator of Differently Transformed GK, EPI and FIB Phenotypes

**Figure 1:**
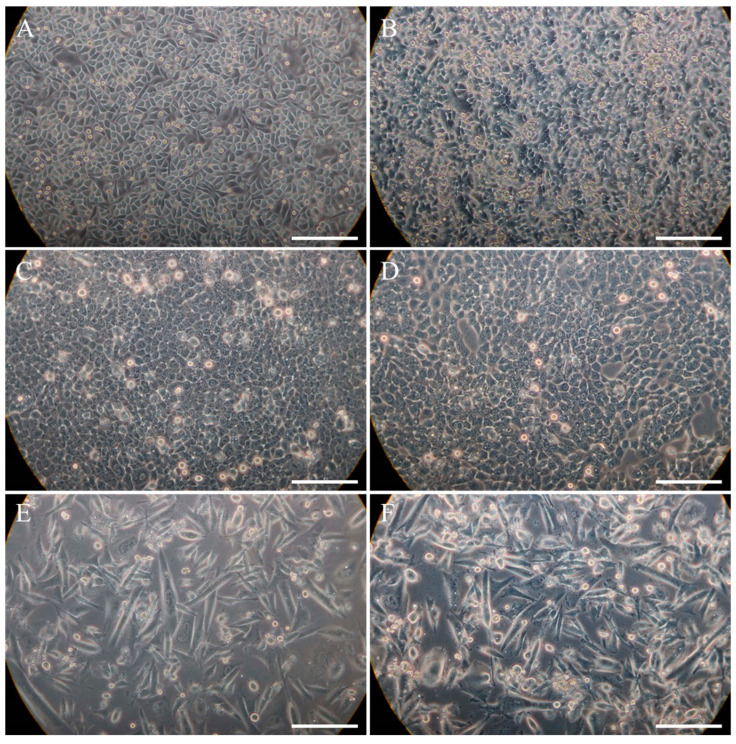
Light microscopic images to visualize the different cell morphologies and degrees of confluence: (**A**) confluent gingival keratinocytes (GKs), (**B**) post-confluent GKs, (**C**) confluent epithelium-like phenotypes (EPIs), (**D**) post-confluent EPIs, (**E**) confluent fibroblast-like phenotypes (FIBs), (**F**) post-confluent FIBs. Bars correspond to 200 µm.

We first monitored the cell morphology in different degrees of confluence to check whether the three phenotypes could be discriminated in terms of their morphological appearance. Analyzing these phenotypes with different physical shapes, we found that cell density generally increased in different confluence levels. However, the phenotypes could be clearly discriminated by their cell morphology and the cell culture per se showed differences as well, which became phenomenologically visible (Figure 1). At confluency, GKs exhibited the typical cobblestone morphology and showed shiny intercellular borders due to low calcium medium conditions (Figure 1A). This fairly applied to EPIs within the confluent stage, whereas cells appeared more granular with less shiny cell-to-cell borders (Figure 1C). By contrast, FIB cells always showed an elongated fibroblast-like morphology, and did not reach the state of a closed cell layer either at confluency or the post-confluence state, the latter defined as 1 week after reaching confluency (Figure 1E,F). While post-confluent EPI cultures contained a high proportion of rounded shiny and thus mitotic cells (Figure 1D), GK counterparts revealed a high frequency of stratified and cornified foci, as indicated by light-dense deposits (Figure 1B). These intra-individual but also culture-associated differences hint that the diversity in the degree of transformation can already be discriminated by phenomenological differences.

### 3.2. Visualization of the Molecules Involved in the Context of Functional Knockdown/Impairment Experiments

**Figure 2:**
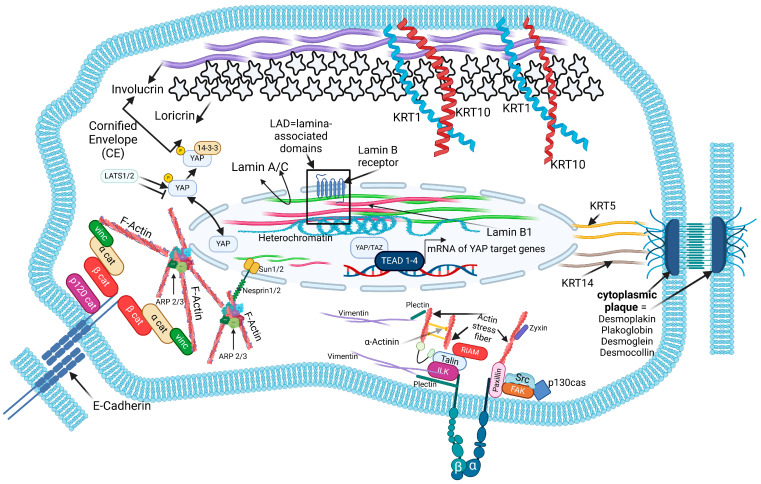
Model cell that visualizes the biomarkers of interest and their molecular context. In addition to the analyzed cIFs and involucrin (IVL), the following molecules were subject of the investigation: CDH1 (E-cadherin), LOR (loricrin), ITGB1 (integrin β1), ITGB3 (integrin β3), LMNB1 (lamin B1), ACTR2 (actin-related protein 2) and YAP1 (yes-associated protein). CDH1 is a constituent of adherens junctions, which connect neighboring epithelial cells to each other, thereby being indispensable for epithelial morphogenesis [48]. Intracellularly, it binds to the actin filament system via linker proteins of the catenin family [49]. In the context of epithelial keratinocyte differentiation in addition to IVL, LOR is a further marker of terminal differentiation and together with IVL participates in cornified envelope formation. LOR can form crosslinks with itself, thereby yielding different molecular weights [40]. Within the lamina-associated domains, the lamin B receptor tethers heterochromatin via LMNB1 to the inner nuclear membrane [50]. After shuttling to the nucleus, the co-transcriptional activator YAP (and its homologue TAZ, neglected in this study) binds to the TEAD family of transcription factors (TEA Domain Transcription Factor1-4), thereby inducing the expression of YAP target genes [51]. At sites of cell matrix interaction, the cIF VIM is involved in the formation of actin-bound FAs via interaction with the FAs constituent plectin [52,53]. Further constituents of FAs are ITGB1 and ITGB3, whereas ITGB3 appears to play a role in the recruitment of VIM and plectin to FAs [54]. Both of the basal cell-specific keratins, the cIFs KRT5 and KRT14, are connected with DP (Desmoplakin) [55], one of the constituents of the cytoplasmic plaque [56]. At sites of adherens junction-mediated cell-to-cell adhesion, CDH1 is linked to the actin cytoskeleton through members of the catenin family and vinculin (VCL) [57]. At sites of the nuclear envelope, ARP2/3 is involved in the formation of the actin cap, which interacts with the inner part of the nucleus through nesprin 1/2 interaction, the latter connected with the nuclear lamins nIFs via members of the Sun-family proteins Sun 1/2 (Sad1/UNC-84 domain-containing proteins) [58]. Created with BioRender.com.

The variety of molecules relevant to the study and the resulting molecular consequences of functional knockdown/impairment experiments inspired us to design a cartoon depicting a model cell that visualizes the biomarkers of interest in their molecular context (Figure 2). The intention was that this model cell should facilitate the reader’s access to impairment-induced modulation of the expression of the biomarkers under study.

The function of the impairment, based on RNAi-mediated knockdowns, affected the cytoplasmic intermediate filaments (cIFs) KRT5 and KRT14 as well as the mesenchymal cIF VIM. In addition, the expression of the terminal keratinocyte differentiation marker IVL was also inhibited by RNAi intervention. Biomarkers used as biological readout for the molecular consequences of the RNAi-based impairment of the expression of the above-mentioned target molecules were those molecules that are involved in the cell and tissue homeostasis of epithelial tissues. In addition to the analyzed cIFs and IVL, the following molecules were subject of the investigation (Figure 2).

Below, in the corresponding subsection, in which the molecular consequences of the specific knockdown of a biomarker relevant to this study are described, the results will not only be presented in continuous text and in tabular form, but will also be made more accessible to the reader using the molecule-relevant image parts of the model cell. In these parts of the image, the up-regulation and down-regulation of molecules affected by the knockdown/impairment of the biomarker are then shown in color-coding. A green marking indicates an up-regulation and a red marking indicates a down-regulation of the expression of the affected molecule at the RNA or protein level.

### 3.3. Basic Molecular Characterization of GKs, EPIs and FIBs Indicates Differential Expression of Biomarkers of Epithelial Tissue Homeostasis

The above phenomenological differences motivated us to investigate putative connections between different morphologies and the expression patterns of biomarkers of epithelial tissue homeostasis in a transformation-related context. In order to later be able to draw a holistic picture of the molecular and functional changes in response to targeted biomarker impairment, we initially examined the three keratinocyte phenotypes in an intervention-free basic setting, i.e., without cIF and IVL impairment (Table 1 and Table 2).

#### 3.3.1. Transcriptional Analysis

Compared to parental GKs, which served as a reference in both the transcription analysis carried out first followed by the protein detection (PCR, Table 1 and Western blot, Table 2), FIB cells showed a drastic down-regulation of the cIFs KRT5, KRT14 as well as KRT1, KRT10 and CDH1. Conversely, they showed a tremendous increase in VIM. Further molecules that reached the level of significance were YAP, which was down-regulated, and integrin ITGB1, which was up-regulated. ITGB3, ACTR2 and LOR in conjunction with IVL were up-regulated, though not reaching the level of significance. Non-significant down-regulation was observed in case of LMNB1. With a focus on EPIs, a transcription-related significance could be demonstrated for the cIFs KRT1, KRT10 and KRT14, but also YAP in the sense of a down-regulation, whereas IVL was up-regulated. The other biomarkers under study revealed both up- or down-regulation at non-significant levels. Among these markers KRT5, LOR, CDH1, ITGB1, LMNB1 and YAP exhibited down-regulation, while ITGB3 and ACTR2 were up-regulated (Table 1).

#### 3.3.2. Protein Expression Detected via Western Blot (WB)

When detecting the protein expression profiles of the biomarkers examined by WB, the very strong expression of VIM was consistent with the gene expression in FIBs. A fairly equal and significant up-regulation of protein was seen for ITGB1. Consistency also applied to the expression levels of KRT1, KRT5 and KRT14 as well as LOR and CDH1, which were significantly lower than in GKs. In contrast to mRNA, no protein was detectable for KRT10 in FIBs. The lack of detection at the protein level was also noticeable for ITGB3 and ACTR2 in FIBs. Non-significant expression differences compared to GK reference cells were detectable in the FIB phenotype for YAP and IVL, the latter of which was up-regulated compared to the previously mentioned molecules. Although not significant at the gene expression level, the LMNB1 protein showed a strongly significant pronounced reduction in FIBs compared with GKs. With regard to EPIs, significantly lower expression levels were detectable for KRT1, KRT14, LOR and CDH1. A lower protein level was also present with respect to LMNB1, although the significance was less pronounced here. Similar to FIB cells, EPI cells also expressed significantly higher levels for the mesenchymal cIF VIM when measured against the GK reference, although the increase in protein levels was not as pronounced as in FIBs. Despite lack of significance, protein levels were increased for IVL and ITGB1 and decreased for KRT5 and YAP. In analogy to FIBs, protein expression for KRT10, ITGB3 and ARP2/3 (ACTR2) was also undetectable in the EPI phenotype (Table 2).

The lack of protein detection for KRT10, ACTR2 and ITGB3 in the GK, EPI and FIB phenotypes may indicate that the protein is not translated or that the translated protein quantity is below the limit of WB detection. The increased or decreased RNA of the above-mentioned biomarkers, seen in EPI and FIBs, may be a first hint of a causal relation to alcohol exposure. Furthermore, the detected gene transcription patterns may indicate that the modulation of transcription of the respective genes apparently occurs at very early transformation stages, since both the EPI and FIB phenotypes are non-tumorigenic in vivo [5].

#### 3.3.3. Fluorescence Imaging to Detect Biomarker Expression and Cellular Topology

**Figure 3:**
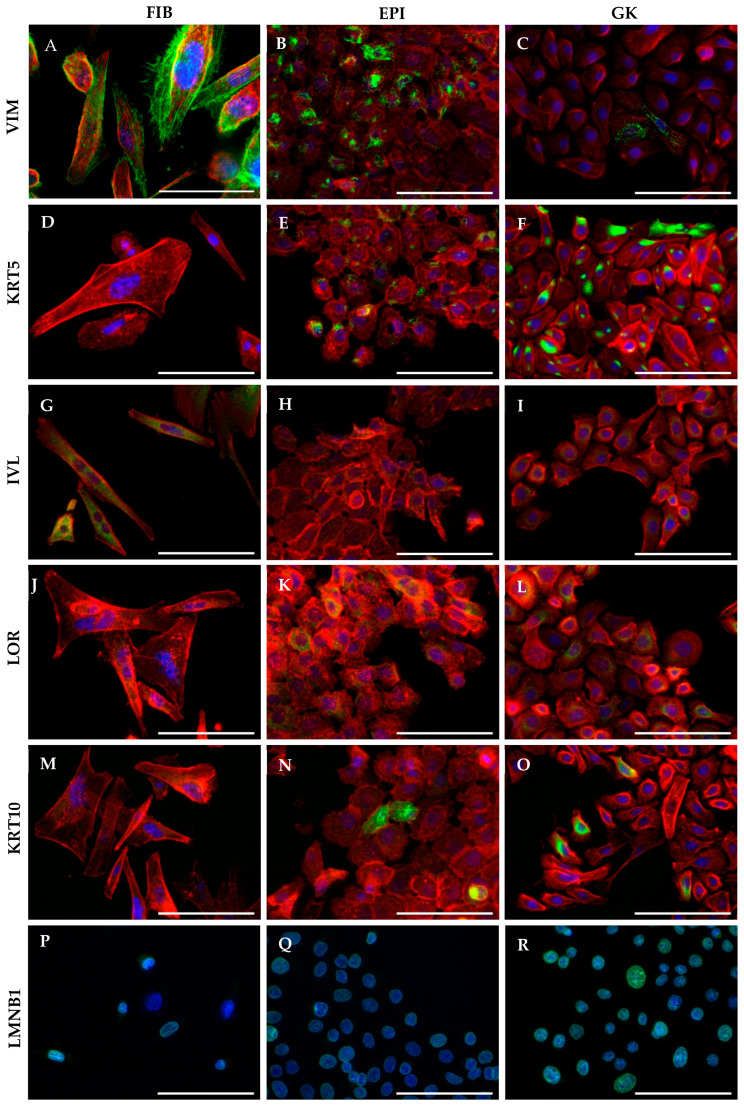
Indirect immunofluorescence of biomarkers under study in GK, EPI and FIB phenotypes of 4-day-old cultures, within the subconfluent culture stage. (**A**–**C**) Vimentin (VIM) expression in all phenotypes with decreasing intensity and changed cellular topology in FIBs (**A**), EPIs (**B**) and GKs (**C**). (**D**–**F**) Cytokeratin 5 staining (KRT5) in FIBs, EPIs and GKs with increasing intensity and different cellular topology. (**G**) Involucrin (IVL) staining in FIBs, (**H**) EPIs and (**I**) GKs with changed cytoplasmic distribution. (**J**–**L**) Loricrin (LOR) staining in FIBs was almost not detectable **(J)** and in EPIs (**K**) and GKs (**L**) with a faint cytoplasmic per-nuclear distribution. Cytokeratin 10 (KRT10) staining in FIBs (**M**) was again almost not detectable and in EPIs (**N**) and GKs (**O**) detectable with an inhomogeneous cytoplasmic distribution. (**P**–**R**) Lamin B1 (LMNB1) staining in FIBs (**P**), EPIs (**Q**) and GKs (**P**) with nuclear localization. Biomarker under study with green fluorescence, red cytoskeleton staining in red and DAPI nuclear counterstain in blue. 60× magnification. Bars correspond to 100 µm.

In addition to the detection of the pure protein amounts, we also focused on imaging to investigate more directly the cell culture-innate topology of the biomarkers under study. Therefore, we carried out immunofluorescence analysis in the GK, EPI and FIB phenotypes of 4-day-old cultures, i.e., within the subconfluent culture stage that is, if the cells have not yet formed a closed cell lawn. For this reason, expression and intracellular immunolocalization are described and shown in high-magnification IIF images (Figure 3), as examples for individual biomarkers under study, and support quantitative protein analyses.

As noted in the previously described WB-baseline analysis, the protein expression of VIM was already significantly increased in EPI cells compared with parental GK cells. This increase over GKs was even more pronounced in FIB cells. This different gradation in VIM protein content is also evident in IIF images of the three phenotypes (Figure 3A–C).

In addition, the fluorescence images also illustrate that in FIB cells, VIM distribution within the cytoplasm formed a homogeneous filament network (Figure 3A). These filamenteous structures were much less present in EPI cells (Figure 3B) and was hardly found in the parental GK phenotype (Figure 3C). Image analysis thus corroborates the quantitative WB-expression findings and suggests, with respect to differential topology, that VIM may be involved in the formation of FAs in FIB cells. This would also be supported by the higher protein levels found in FIBs for ITGB1 (Table 2) [53,54].

Regarding KRT5, fibroblastoid FIB cells were largely negative with respect to specific green fluorescence (Figure 3D), thereby suggesting only residual expression. In contrast, KRT5 distribution in EPI cells occurred in a punctate patchy pattern, and the entire culture was not homogeneously positive (Figure 3E). A cellular much more extensive KRT5 signal with almost homogeneous distribution in the culture was visible in parental GK control cells (Figure 3F). Consistent with the baseline, the FIB and EPI phenotypes were largely negative for KRT14, whereas GK cells showed a distribution pattern fairly comparable to KRT5.

For the early and terminal differentiation markers KRT10 as well as IVL and LOR, FIB cells showed an unexpected pattern especially for IVL. Contrary to the assumption that cells, morphologically more resembling mesenchymal fibroblasts, they showed culture-inherent homogeneous cytoplasmic IVL green fluorescence (looking partially yellow in the red actin merge) (Figure 3G), which occurred to a much lesser extent in the EPI and GK phenotypes (Figure 3H,I). An inverse picture emerged for LOR, as here FIB cells were almost negative (Figure 3J), whereas EPI and GK cells showed a faint perinuclear expression (Figure 3K,L). The discrepancy between mRNA and protein for KRT10 was also evident in IIF in agreement with baseline WB analysis. Here, FIB cells showed only the red fluorescence for the actin cytoskeleton (Figure 3M), whereas in EPIs and GKs (Figure 3N,O), KRT10 expression was inhomogeneously distributed in the cytoplasm. This discrepancy may indicate that the significantly lower amount of KRT10 mRNA compared with GKs suggests a gene expression that is no longer translationally relevant at the protein level.

When looking at nIFs and here LMNB1, which is relevant for this study, it was noticeable that compared to GK parental cell cultures (Figure 3R), the nucleus-associated green fluorescence in EPI cells (Figure 3Q), although homogeneously distributed in the culture, already started to decrease (compare Figure 3R with Figure 3Q) and continued to decline further in FIBs (Figure 3P). In conjunction with the LMNB1-WB baseline results, which reveal a progressive decline in EPIs and FIBs, this could indicate changes in nuclear integrity. This notion will be supported by the abundance of pyknotic cell nuclei in FIB cells irrespective of VIM knockout, since pyknotic nuclei are fairly equal in percentage in the non-targeting (nt) control (Figure 5). Therefore, it seems to be a connection between LMNB1 nuclear integrity and pyknotic cell nuclei in our cell system, which may point to the existence of apoptotic events.

### 3.4. Molecular and Cell Functional Consequences of Selective Biomarker Impairment

A basic requirement following application of siRNAs, the respective cIFs and IVL in the three phenotypes were significantly inhibited at the RNA and protein level, respectively. The results indicating the percentage of inhibition can be found in detail in Table 3 and Table 4.

#### 3.4.1. Molecular Consequences in the Course of RNAi-Mediated Gene Expression Impairment

The so far presented data of the basic characterization paint a picture in which molecular differences including cIFs like KRT5, KRT14, and VIM become evident in dependency of the cell transformation stage of GKs, EPIs and FIBs. However, the functional and cell behavioral consequences of cIF impairment, which will shed light on the decisive role of these cIFs in maintaining proper cell and tissue physiology is unclear in our cell system. To start answering this question, we detected the expression status of the biomarkers under study on both the gene and protein expression level in response to RNAi-based impairment of KRT5, KRT14 and IVL. Although the knockdown-related modulations of the investigated biomarkers can be seen in detail in Table 3 (*mRNA expression*) and Table 4 (*protein*), we would like to comment on some very interesting examples from our point of view below with reference to the cartoon (see Figure 2).

**Figure 4:**
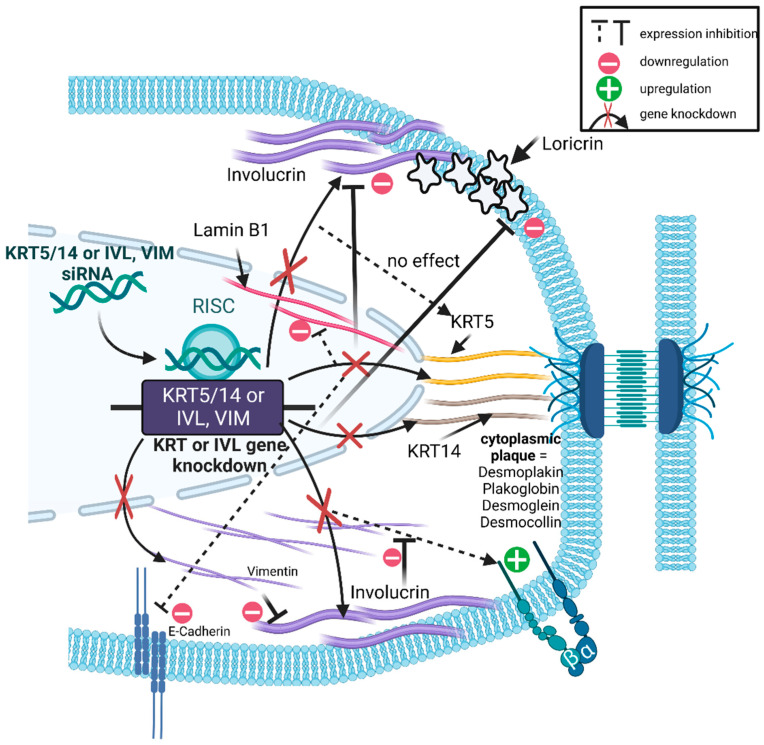
The cartoon illustrates based on Figure 1 the used gene knockdowns/impairments and their respective effect on biomarkers under study. The figure shows in detail the RNAi-based intervention of KRT5/14, IVL and VIM, and their upstream and downstream effects in the cell. It shows either the up-regulation of the expression with a plus sign or the down-regulation with a minus sign of the corresponding genes. Created with BioRender.com.

One of the unexpected examples is the reciprocity between the cell-specific keratins and the terminal differentiation-indicating cornified envelope component IVL [40], which manifested itself at least at the RNA level. Here, the impairment of KRT5 and KRT14 mostly caused a down-regulation of IVL gene expression. Conversely, the detected RNA levels of basal cell-specific keratins were decreased when IVL was inhibited (Table 3, Figure 4). At the protein level, the relationship between KRT5, KRT14 and IVL was unilateral, that is, impairment of both KRTs usually inhibited IVL at the level of significance, but not vice versa (Table 4). Another unforeseeable correlation could largely be determined for the mesenchymal cIF VIM and IVL, which was also integrated into our studies. The mutual influence was often significant and revealed itself in a down-regulation of the respective partner. This down-regulation affected both levels of detection, including RNA (Table 3, Figure 4) and protein (Table 4, Figure 4). Another molecule in the biomarker portfolio examined that was affected by the VIM impairment was ITGB1. Here, increased protein levels were found after VIM intervention in all three phenotypes, with the increase in FIBs and EPIs showing a level of significance (Table 4, Figure 4). In the keratin context, it was also observed that as a result of the RNAi intervention, there was often a significant reduction in the protein amount of the second cornified envelope constituent examined, LOR [40], with regard to both KRT5 and KRT14 (Table 4, Figure 4). On the gene expression level, this significant reduction became feasible exclusively for KRT5 in EPI cells. However, though not reaching significance, a lower amount of LOR transcripts was also found in FIBs (Table 3), suggesting a slight predominance of KRT5 in the context of terminal differentiation in the employed cell system. The assumed probability of a dominant role for KRT5 in the epithelial differentiation process continues when considering the status of LMNB1, a molecule that plays an important role in maintaining nuclear integrity [59]. Although significantly increased at the mRNA level in FIB cells (Table 3), the KRT5 impairment at the protein level led to a mostly very pronounced reduction in the LMNB1 protein, regardless of the respective phenotype (Table 4, Figure 4). In addition, KRT5 intervention also had negative consequences on adherens junctional CDH1 [60], which was clearly expressed in GK, but also in transformation-advanced EPI cells (see Table 3, as well as Table 4). In both phenotypes, the negative consequences were represented by a substantially reduced protein presence of CDH1 (Table 4, Figure 4).

#### 3.4.2. Gene Impairment-Related Consequences on Proliferation

The results so far indicate that there are expression-related interactions between epithelial terminal differentiation and the analyzed cIFs, which in the case of the basal cell-specific keratins KRT5 and KRT14 affect the proliferative epithelial area. Against this background, we next asked whether the RNAi-induced impairment of the investigated biomarkers KRT5, KRT14 and VIM as well as IVL also has cell functional consequences. For this reason, we analyzed proliferation because, in addition to terminal differentiation, it represents another key pillar of tissue homeostasis [61] and thus physiology. As can be seen from Table 5, the proliferation analysis revealed an inhibition of proliferation that was largely independent of cell transformation and biomarkers. The only exception to this generalization was the impairment of VIM in the GK phenotype, which resulted in consistent marginally increased proliferation (see Table 5). In the case of the KRT5 knockout, significant inhibition was observed for the FIB and GK phenotypes, whereas inhibition in EPIs fell just short of significance (see Table 5). With regard to proliferation inhibition, further significance was detectable for biomarker impairments VIM and IVL in EPIs and KRT14 in GKs (see Table 5). These findings show that the cIFs examined, but also the terminal differentiation marker IVL, are involved in some way in the regulation of proliferation and, in the cIF investigation context, indicate that each individual intermediate filament has relevance for cell and tissue physiology.

#### 3.4.3. Nuclear Pyknosis and cIF-Impairment-Related Chromatin Changes

**Figure 5:**
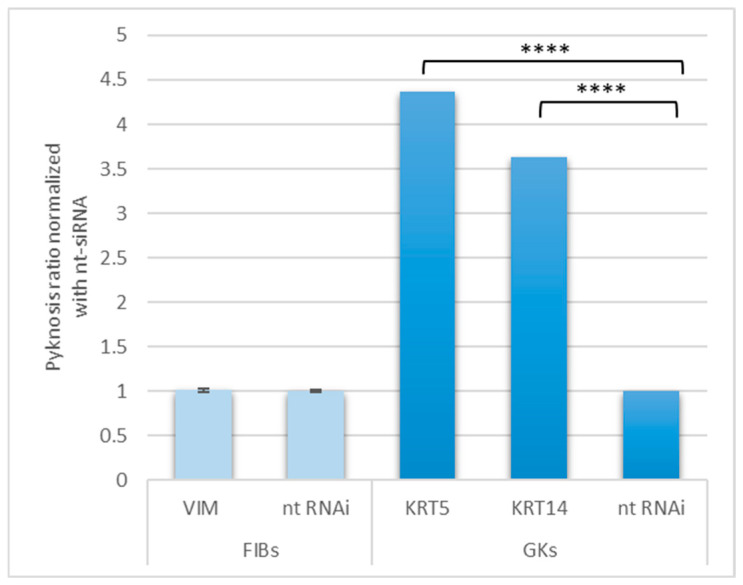
Consequences of selectively evaluated impairments of KRT5, KRT14 and VIM on the proportion of pyknotic nuclei in FIBs and GKs. The total number of DAPI-stained cell nuclei was compared with the number of pyknotic cell nuclei, statistically analyzed using an unpaired *t*-test and compared with the nt-siRNA control (n = 3, mean value ± SEM, *p* < 0.0001 ****).

The LMNB1-related differences within the phenotypes as such and the cIF-impairment-related LMNB1 alterations (see Table 3 and Table 4) may imply possible effects on the structure of the cell nucleus. This is because LMNB1, as an nIF, is involved in maintaining cell nuclear integrity [62]. This connection inspired us to next look for possible impairment-related changes in nuclear shape. In order to be able to show a possible dependence on the degree of transformation of the cells, we carried out this analysis in GK and FIB cells, since FIB cells are already more advanced in cell transformation compared to the EPI and parental GK cells. To clarify the role of the cIFs in particular, we focused on VIM, which is predominant in FIB cells, and on the basal cell-specific cIFs KRT 5 and KRT14 in GKs. The results, which are depicted in Figure 5, revealed a fairly equal percentage of pyknotic nuclei in the VIM non-targeting(nt) control, irrespective of VIM impairment. This finding may indicate that FIB cultures per se have a very pronounced nuclear pyknosis, and further supports the notion that LMNB1 may be involved herein. This involvement appears plausible, since FIB cells basically express LMNB1 significantly lower than GK, independent of cIF-impairment (see also Table 3 and Table 4). In GKs, the intervention of both basal cell-specific cIFs led to a significant increase in nuclear pyknosis (Figure 5). Interestingly, only the KRT5 impairment and not that of KRT14 leads to a reduction in LMNB1 expression (Table 4). This result supports the notion that KRT14 indirectly increases putative LMNB1-associated nuclear pyknosis via KRT5, and that KRT 5 plays a more important role here. This is because the RNAi intervention of KRT14 significantly inhibits the expression of its filament binding partner KRT5 (Table 4). Since nuclear pyknosis reflects a final stage of cell functional apoptosis, we next searched for indications of apoptotic events by fluorescence imaging of caspase-3, known as an executioner proteolytic protease, which triggers the final stages of programmed cell death [63]. In fact, we detected stronger fluorescence caspase 3 signals in GK cultures subjected to KRT5 and KRT14 impairment (Supplemental Figure S1). This result provides further evidence that cIFs KRT5 and KRT14 in particular in addition to proliferation are directly or indirectly involved in further cell functions such as apoptosis.

Since LMNB1 is an essential organizer of nuclear chromatin [64], it was obvious to us to investigate whether the above-mentioned cIF impairments also cause changes in the status of the chromatin in the GK and FIB phenotypes. To obtain information about this issue, we treated cells with eu- and heterochromatin-specific antibodies after extraction of the histone fraction from RNAi-treated versus nt control cells. This selectively discriminated transcriptionally active eu-, H3K4me3 and H3K9 from transcriptionally inactive heterochromatin, H3K9me3 and H3K27me3 [36,65]. Interestingly, all cIF impairments in the examined phenotypes led to a modulation of the eu- and heterochromatin-indicative histone H3 markers (Table 6). Regarding the euchromatin marker H3K9ac, the VIM intervention in FIB led to a significant reduction, which also applied to the other markers in a non-significant form (Table 6). H3K9ac was also significantly reduced in GKs in response to KRT5 impairment, and remained fairly constant after KRT14 intervention. In light of the VIM and KRT5-related lamin B1 protein reduction in FIBs and GKs, described before (Table 4), the euchromatin-indicative reduction in H3K9ac may possibly be associated with the decrease in LMNB1. Non-significant modulation of the other histone H3 markers examined indicated reduced levels in GKs upon KRT5 impairment for H3K4me3 (eu-) and increased levels for H3K9me3 and H3K27me3 (heterochromatin). In the case of the KRT14 impairment, with the exception of H3K27me3 (heterochromatin), all other histone H3 markers were not significantly increased (Table 6). The partially significant modulation of the histone H3 modifications examined shows that the status of both euchromatin and heterochromatin is changed by the impairment of specific cIFs in our cell system. These chromatin changes may be a first indication that the studied cIFs influence the cellular transcriptome in a broader sense.

## 4. Discussion

Here we identified molecular consequences of selective RNAi-based cIF and IVL impairment in a unique oral keratinocyte cell system, which represents advanced cell transformation stages in response to alcohol treatment. In the course of this alcohol treatment, among other things, a stable phenotype has been established which, regardless of its molecular composition, corresponds at a morphological level to the fibroblast-like morphology typical of EMT. With its morphology, this phenotype, which we call FIB, meets the criteria catalog for EMT cells, which is described in the review article “Guidelines and definition for research on EMT” by Yang et al. [66]. Moreover, these FIB cells showed a significant increase in SNAIL1 and ZEB1 expression (Supplemental Figure S4), whereas SNAIL1 has been linked earlier to triggering EMT during tumor progression [67]. While at the molecular level the increase in expression of VIM is in accordance with the previously mentioned guidelines, the increase in IVL in EPIs and FIBs detected in our cell system is not described in the guidelines and therefore represents an incidental finding. In connection with the protein expression of IVL in EPI and especially FIB cells, it seems worth mentioning that the data in the WB analysis are not significant, due to the biological heterogeneity. But the protein bands detectable in the WB are clearly stronger in EPI and even stronger in FIB than in GK reference cells (Supplemental Figure S2). Since rose absolute oil (RAO), an essential oil containing phenethyl alcohol as the main component, has been shown to increase IVL expression in skin keratinocytes, the induction in EPIs and FIBs may possibly be due to alcohol treatment of the cells and potentially a specific feature of alcohol-associated EMT [68].

Further, the results revealed an unexpected interdependence between one of cIFs and IVL and vice versa. We perceived distinct elements for discussion. To start, we showed that impairment of KRT5, KRT14 and VIM but also IVL yields molecular consequences that affect the expression of biomarkers relevant for proper cell and tissue physiology as mirrored by detection of their up- and down-regulation on both the gene but also the protein expression level. In the analyzed epithelial phenotypes, three cIFs typically expressed under in vitro conditions were addressed in an RNAi-based interventional manner, leaving it to future studies to identify and characterize the molecular and cell functional consequences of additional epithelial cIFs. Irrespective of this, our results further show that RNAi-based impairment of the corresponding cIFs and IVL has consequences not only on the molecular level but apparently also on cell functions, as is evident from the findings for proliferation and differentiation, nuclear pyknosis and transcriptome status.

The results are seminal for further uncovering the role of specific molecules in the context of lifelong maintenance of cell and tissue physiology. This is because, as shown here for the cIFs KRT5, KRT14 and VIM, there is a reciprocal influence between them and IVL either at both or at least one of the levels of evidence, i.e., RNA or protein. This reciprocity reveals the complexity of the interaction of biomolecules in the context of maintaining cell and tissue physiology. Furthermore, we were able to show for the first time that regardless of the degree of transformation, i.e., in all three keratinocyte phenotypes, in addition to basal cell-specific KRT14, KRT5 is apparently also causally involved in epithelial terminal differentiation per se. This is because in the EPI and parental GK phenotype, KRT5 RNAi intervention leads to a significant modulation of LOR expression. However, in addition to this transformation-independent causality, the modulation as such is most likely transformation-dependent, as KRT5 impairment results in increased expression of LOR in GKs and decreased expression in EPIs. Although in FIB cells it is not KRT5 but KRT14 intervention that causes decreased LOR expression, this finding nevertheless supports the obvious suggestion that the basal cell-specific cIFs KRT5 and KRT14 are involved in the regulation of LOR in a transformation-dependent manner. This is an important point because even in oral epithelia, LOR as a terminal differentiation marker accounts for more than 70% of the cornified envelope and thus is actually even more involved than IVL in maintaining epithelial barrier function of the stratum corneum [69]. In parental GK cells, the enhancement of LOR expression by KRT5 intervention suggests that this cIF is involved in the control of terminal differentiation, which represents a cornerstone of epithelial tissue homeostasis and thus tissue physiology [61]. In EPI and GK cells, KRT5 impairment-related LOR decrease indicates that when epithelial cells adopt more and more the EMT phenotype, normal epithelial terminal differentiation is disrupted. This is mainly described in EMT-associated studies, which usually reveal IVL decrease [70,71]. However, our data elaborated in FIB cells support the suggestion that disturbances in terminal differentiation in response to basal cell-specific KRT-defaults may occur already in a *pre* EMT stage of cell transformation. This suggestion is backed up by the finding that in both phenotypes, namely GKs and EPIs, KRT5/KRT14 intervention yields significantly decreased IVL protein levels. While literature findings on the situation regarding KRT14 depletion are available, the data regarding the molecular and cell functional consequences of an intervention against KRT5 are very sparse or, better yet, not available. In this context, Alam et al. describe for an oral squamous cell carcinoma cell line (AW13516 from the tongue) that the KRT14 impairment led to an increase in IVL expression and further show that the KRT14 intervention led to a significant loss of KRT5 [72]. In our system, this possible regulatory effect of KRT14 on its basic partner KRT5 applies to all three phenotypes at the gene expression level, whereas only the GK phenotype is affected at the protein level. This, like the discrepancy in IVL expression between our system and the findings of the Alam group, may possibly be due to the degree of transformation of the cells examined or their tissue origin. The aspect of the tissue origin of the cells under consideration can be taken into account because, in addition to the AW13516 cells, Alam et al. also examined the spontaneously immortalized keratinocyte cell line HaCaT, which, with analogous findings regarding the consequences of the KRT14 knockdown, comprises epidermal skin keratinocytes [72].

As already mentioned in the introductory remarks of the discussion, there is a correlation between cIFs and IVL, which applies to VIM from the perspective of cIFs. In this context, it is worth mentioning that this correlation also applies to parental GK cells, since sporadic expression of VIM was still detected using IIF in GK cultures independent of WB. While the relationship between VIM and IVL has been published, there are no literature references regarding the influence of IVL on VIM. An up-regulation of IVL after RNAi-based VIM depletion was found in malignantly transformed oral cancer cells [73]. This up-regulation is not consistent with our data revealing significant down-regulation of IVL following VIM depletion. This discrepancy may also be an indication that the expression patterns of interesting endpoint molecules, in this case IVL, depend on the transformation stage of the cells examined. This is because the cells in the Dmello study have already been transformed into malignant cells, i.e., they represent derivatives of an oral squamous cell carcinoma (OSCC) and thus represent the final stage of tumor progression-related transformation. However, with the immortalized oral GK phenotype, our cells are in the earliest detectable stage of transformation and EPI and FIB cells can be viewed as more advanced stages due to their molecular makeup, but are demonstrably not yet tumorigenic [5]. In particular, the FIB phenotype shows signs at the molecular level that usually characterize the EMT phenotype, which is why the FIB phenotype of our cell system can be considered the most advanced in cell transformation [6].

Furthermore, our data support the conception that residual expression of a biomarker in cells may also be sufficient to maintain important cellular functions. An example is found in the expression of KRT5 in FIB cells, whose gene expression is very weak compared to GKs and whose protein is undetectable, which may be due to quantity detection limits of WB. Despite this limitation, impairment of KRT5 in FIB cells results in significant down-regulation of the LMNB1 protein, a molecule involved in maintaining nuclear integrity [74]. This role of KRT5 in nuclear integrity is supported by the findings in EPI and GK cells, where KRT5 impairment also causes significant inhibition of LMNB1 expression. A role for KRT5 in LMNB1 regulation can be derived from two publications that focus on the transcription factor p63. First, Romano et al. describe that the Δ isoforms of the transcription factor p63 (ΔNp63) have an active role in regulating basal keratin genes KRT5 and KRT14 [75]. Second, Rapisarda et al. have found that p63-depleted mice and derived keratinocyte in vitro cultures exhibit nuclear abnormalities and reduced LMNB1 expression as well as chromatin modulation-associated down-regulation of transcription [76]. Although not analyzed in detail, against the background of the findings regarding KRT5/14, p63 and LMNB1 described above, there is the possibility that there is an analogy to our cell system. This analogy especially holds true for KRT5, since here too the impairment-related KRT5 down-regulation leads to a significant reduction in LMNB1 protein expression, which can partly also be seen at the gene expression level. This could also be a possible explanation for the increase in pyknotic-apoptotic cell nuclei in the context of impairment of basal cell-specific keratins, which will be discussed later.

Another work by Truong et al. [77] shows even more extensive functions of the transcription factor p63 in connection with stratified epithelia. In organotypic in vitro cultures of normal human keratinocytes, they were able to show, among other things, that p63 knockdown via siRNA inhibits proliferation, stratification and early epithelial differentiation (indicated by KRT1/10 expression). Against this background, it can be speculated that signaling events involving p63 may not only directly or indirectly address KRT5 or KRT14, but also proliferation, since in all of our three keratinocyte phenotypes impairment of KRT5 commonly leads to a significant reduction in proliferation (Table 5). This speculation is backed up by findings elaborated by Srivastava et al., who found that KRT14 knockdown leads to increased amounts of the TAp63 isoforms on both the gene as well as the protein expression level [12]. Although these isoforms are less responsible for proliferation than the ΔNp63 isoforms and are mainly responsible for epithelial differentiation, the results of the Svaristava study show that basal cell-specific keratins are involved in the regulation of transcription factors that control epithelial physiology. Therefore, it cannot be excluded that knockdown of basal cell keratins also affects the ΔNp63 isoforms. Regardless of the connections between p63, KRT5, KRT14 and proliferation just discussed, it is worth mentioning that an inhibition of proliferation because of KRT14 knockdown could be observed in epithelial cells [72]. Such inhibition was also significant in the case of the GK phenotype with regard to the KRT14 intervention (Table 5). Further evidence of a connection between the basal cell-specific cIFs and proliferation arises from the situation of the potentially active YAP detected in the cell nuclear fractions because of knockdown (Table 5). Here, when KRT5 is impaired in GKs and KRT14 in FIBs, there is a significant reduction in nuclear YAP. In both cases, this coincides with a reduction in proliferation, which is also significant in GKs for KRT5 (see Table 5). Of note, the above-mentioned connection between YAP and proliferation is based on literature data that have identified YAP target genes that stimulate proliferation, such as cyclin D [78].

Another cIF that, in addition to KRT5 and KRT14, apparently plays a role in proliferation regulation appears to be VIM. This is because, at least in the FIB phenotype, there is a significant reduction in proliferation because of VIM depletion (Table 5). Although very thin, there is evidence in the literature that the knockout of VIM has negative consequences on proliferation, which is so important for tissue physiology. This has been demonstrated in a work on fibroblasts in the context of wound healing [32] and murine embryonic stem cells [79]. The previously described finding of reduction in proliferation in FIBs as a result of VIM intervention supports the notion that VIM is involved in the control of proliferation in cells that are a little more advanced in cell transformation and in the process of adopting an EMT-like phenotype.

Another, from our point of view, extremely unexpected molecule that, in addition to VIM, inhibits proliferation in FIB cells is the terminal differentiation marker IVL (Table 5). Although it was not the aim of the present work to investigate the molecular mechanism for this issue, the following consideration described below may provide a possible explanation. In previous studies, we were able to show that epithelial cells express increased differentiation when they are exposed to cell-damaging influences; in our case, these were remaining monomers from dental plastics [80]. Keratinocytes that differentiate soon leave the epithelial network so that the stimulus for proliferation is given in order to maintain the epithelial network in the sense of physiology [8,39]. The increased abundance of IVL in EPI cultures can therefore represent such a proliferation stimulus, which is then no longer present due to the intervention and leads to INV impairment-related proliferation inhibition.

Based on CDH1 expression, our cell system further illustrates that KRT5 probably plays an important role in maintaining normal epithelial tissue physiology. This is because KRT5 ablation in GK and EPI causes a significant decrease in the CDH1 protein, which is involved in the formation of adherens junctions in squamous epithelia. Such a causal link between KRT5 knockdown and loss of CDH1 has been demonstrated under in vitro conditions for basal-like breast cancer cells [81], i.e., cells that have already undergone malignant transformation, which, as already mentioned, represents the final stage of the entire transformation process. However, our findings from GK and EPI cells show that this down-regulation of CDH1 caused by KRT5 inhibition is not a phenomenon of the final tumor-related cell transformation, i.e., the malignant tumor in vivo, but rather occurs at the beginning of the transformation process, i.e., if the cells are not yet tumorigenic. Such facts, developed through our studies, not only contribute to expanding knowledge about the function of cIFs in epithelial cells and tissues, but can also serve to identify and characterize new biomarkers in the context of early cancer detection and prevention.

Another example that putative residual protein expression may be biologically relevant can be found in our study of INTB3. The significant increase in INTB3 gene expression during VIM impairment in EPIs and FIBs, given relatively constant ITGB1 gene expression, can be an indication that the cells compensate for the lack of VIM by increasing INTB3 transcription in order to be able to recruit remaining amounts of VIM to the FAs. This explanation seems plausible because Burgstetter et al. describe that ITGB3 is probably involved in the recruitment of VIM in conjunction with plectin into FAs and is therefore beneficial for the stabilization of FAs [54]. In this context, the significantly increased protein expression of ITGB1 in EPIs and FIBs (GKs exhibit non-significant increase) would also make sense, as the cells rely on FAs to maintain adhesion and also migration. Regarding migration, this FAs requirement applies especially to EPIs and FIBs, since the FIB phenotype in particular shows transformation progression-indicative EMT-associated characteristics. Another finding supporting the increase in ITGB1 in our system is that VIM is important for ITGB1 trafficking to the leading edge of migrating carcinoma cells [30], which have completed the process of cell transformation. As already discussed for INTB3, it may be possible that in EPIs and particularly FIBs, the increased ITGB1 expression upon VIM impairment represents a compensatory measure by the cell to ensure the maintenance of FAs.

In addition to the molecular consequences of the KRT5 intervention on LMNB1 discussed above, the impairment of VIM also has very negative effects on LMNB1 expression, especially in EPIs and GKs (Table 4). From work on mouse embryonic fibroblasts with wild-type and mutant VIM, it appears that VIM is involved in maintaining nuclear stability [82]. Moreover, it has been shown that loss of K14 leads to nuclear anomalies [83] and loss of K1 and K10 causes disruptions in nuclear integrity [84].

These literature findings show that the cIFs examined in the present work are important for nuclear integrity. Regarding our cIF knockdown-related LMNB1 reduction, published findings show that LMNB isoform and LMNB1 loss are associated with nuclear deformations like blebs or speckles [36,85]. In this context, it is important to point out that bubbling is frequently related to nuclear instability [86]. This literature evidence suggests that the cIF knockouts found in our system, i.e., KRT5, KRT14 and VIM, can also lead to an alteration in cell nuclear morphology associated with LMNB1 down-regulation. In fact, we were able to demonstrate increased nuclear deformation in the form of nuclear pyknoses, particularly in the FIB phenotype, which did not increase further even after VIM intervention. This may be because the FIB phenotype per se already has significantly lower LMNB1 expression than the GK phenotype (Table 2). Despite the significantly lower amount of LMNB1 protein compared to the GK cells, the VIM impairment still leads to a further, although not significant, down-regulation of LMNB1 in FIB cells (Table 4). This suggests that VIM can be involved in the cellular abundance of LMNB1 regardless of the per se content of LMNB1, and irrespective of whether VIM is strongly or residually expressed, as is the case in GK cells. The published involvement of basal cell-specific keratins in cell nuclear deformation [83] could also be verified in our system, as KRT5 and KRT14 impairment led to a significant increase in pyknotic nuclei in GK cells (Figure 5). The reduced levels of LMNB1 after cIF knockdown, known to be related to apoptosis [37], and the associated pyknotic nuclear deformations suggest that apoptotic processes may also occur in the corresponding cell entities. This assumption is supported by the fact that pyknotic cell nuclei represent late stages of nucleus-related apoptosis because of chromatin condensation [87]. In fact, in cultures with severe nuclear pyknosis, fluorescence imaging revealed slightly stronger signals for caspase 3, which was particularly true for the GK phenotype after KRT5 and KRT14 intervention (Supplemental Figure S1). This suggests that basal cell-specific cIF impairment may serve as an apoptotic trigger. In addition to the importance of LMNB1 in nuclear integrity, another facet of this molecule is that it appears to be involved in proliferation. This emerges from work that showed that loss of LMNB1 leads to an extension of S phase, resulting in S-phase accumulation of cells and subsequent reduction in proliferation [35]. In our system, there is evidence for this connection, as LMNB1 is reduced by KRT5 and VIM impairment and the cIFs mentioned lead to a significant proliferation inhibition after intervention.

It is known that DNA–lamin interactions at the nuclear membrane determine the function and organization of chromatin. In this connection, LMNB1-associated domains are characterized by heterochromatin-related low gene expression levels [36,88]. Since we were able to show a connection to LMNB1 expression, particularly for VIM and KRT5, we examined the chromatin status using specific histone modifications for eu- and heterochromatin. We detected a partially significant up- or down-regulation of the eu- and heterochromatin-indicating histone markers as a result of the impairments, carried out for KRT5 and KRT14 in GK and VIM in FIB cultures. This shows that the interventional influence on cIF expression affects the status of the chromatin and in a broader sense may influence the transcriptome of the analyzed phenotypes.

## 5. Conclusions

Our novel findings suggest that many of the changes detected by the RNA-based impairment of cIFs and IVL are related to the degree of cell transformation. Further, they indicate that many of the molecular and cell function-related changes already take place in very early, non-tumorigenic stages of carcinogenesis, i.e., in those in which the cells are on the way to adopt the EMT phenotype. In addition, our experimental findings illustrate the functional importance of basal cell-specific cIFs and especially KRT5, but also VIM and IVL, for the maintenance of such important physiological cell functions as epithelial differentiation and proliferation as well as apoptosis. They also provide initial evidence that the molecular and cell functional changes detected in the course of the specific impairments are associated with a modulation of the transcriptome. This knowledge extension in the field of epithelial cIF and IVL functions in health and disease is of great benefit, since it can serve to identify and characterize new biomarkers in the context of early cancer detection and prevention.

## Figures and Tables

**Table 1:** Different expression between GKs, EPIs and FIBs of individual markers at RNA level using qPCR (high standard deviations within the replicates prevent significance for some markers, despite high values).

qPCR	Mean Values						
	FIB		EPI		GK		
**VIM**	1713.16 ****		32.13 ns		1.00		
**KRT5**	0.00 ****		0.59 ns		1.00		
**KRT14**	0.01 ****		0.24 **		1.00		
**KRT1**	0.01 ****		0.06 ****		1.00		
**KRT10**	0.14 ****		0.33 *		1.00		
**IVL**	144.98 ns		5.74 *		1.00		
**LOR**	2.07 ns		0.97 ns		1.00		
**CDH1**	0.00 ****		0.56 ns		1.00		
**ITGB1**	4.59 **		0.67 ns		1.00		
**ITGB3**	80.17 ns		3.01 ns		1.00		
**LMNB1**	0.60 ns		0.72 ns		1.00		
**ACTR2**	1.56 ns		1.01 ns		1.00		
**YAP1**	0.34 ***		0.51 ns		1.00		
Expression	>10	>5	>1	=1	<1	<0.5	<0.1
*p*-Value	ns = not significant		* = *p* < 0.05	** = *p* < 0.01	*** = *p* < 0.001	**** = *p* < 0.0001	

**Table 2:** Different expression between GKs, EPIs and FIBs of individual markers at protein level using Western blot (high standard deviations within the replicates prevent significance for some markers, despite high values).

Western Blot	Mean Values					
	FIB		EPI		GK	
**VIM**	8369.14 ***		4.05 *		1.00	
**KRT5**	0.00 ****		0.93 ns		1.00	
**KRT14**	0.00 ****		0.00 ****		1.00	
**KRT1**	0.15 ****		0.56 ****		1.00	
**KRT10**	-		-		-	
**IVL**	2154.02 ns		282.21 ns		1.00	
**LOR**	0.51 ***		0.48 ****		1.00	
**CDH1**	0.00 ****		0.71 **		1.00	
**ITGB1**	18.68 *		1.55 ns		1.00	
**ITGB3**	-		-		-	
**LMNB1**	0.49 ***		0.60 **		1.00	
**ARP 2/3**	-		-		-	
**YAP**	0.91 ns		0.79 ns		1.00	
Expression	>10	>5	=1	<1	<0.5	<0.1
*p*-Value	ns = not significant		* = *p* < 0.05	** = *p* < 0.01	*** = *p* < 0.001	**** = *p* < 0.0001

**Table 3:** Consequences of the individual RNAi interventions in GK, EPI and FIB cells on various residual RNA activities by qPCR, presented in mean values (MV) and *p*-values.

qPCR		FIBs					EPIs					GKs				
		KRT5-RNAi	KRT14-RNAi	IVL-RNAi	VIM-RNAi	nt-RNAi	KRT5-RNAi	KRT14-RNAi	IVL-RNAi	VIM-RNAi	nt-RNAi	KRT5-RNAi	KRT14-RNAi	IVL-RNAi	VIM-RNAi	nt-RNAi
**MV**	**KRT5**	0.62 ns	0.26 **	1.17 ns	2.04 ns	1.00	0.01 ****	0.63 *	0.45 ***	0.59 **	1.00	0.02 ****	0.46 *	0.66 **	0.55 ****	1.00
	**KRT14**	1.19 ns	1.28 ns	0.31 ns	1.31 **	1.00	0.21 ***	0.49 **	0.38 ***	0.48 ****	1.00	0.77 ns	0.03 ****	0.89 ns	0.92 ns	1.00
	**VIM**	1.16 ns	0.96 ns	0.87 ns	0.03 ****	1.00	0.81 ns	0.69 *	0.56 ****	0.01 ****	1.00	0.71 ns	0.92 ns	0.37 ****	0.05 ****	1.00
	**IVL**	0.76 ns	0.74 *	0.02 ****	0.72 ns	1.00	0.58 ns	0.38 **	0.01 ****	0.12 ****	1.00	1.29 ns	0.19 ***	0.03 ****	0.34 ****	1.00
	**LOR**	0.81 ns	1.35 ns	0.30 ns	0.47 **	1.00	0.23 *	0.69 ns	0.66 ns	0.75 ns	1.00	1.74 ns	1.56 ns	1.51 ns	1.54 ns	1.00
	**CDH1**	1.30 ns	1.42 *	0.56 ns	0.89 **	1.00	0.96 ns	0.77 ns	0.87 ns	0.41 ****	1.00	1.55 ns	0.79 ns	1.30 **	0.71 *	1.00
	**ITGB1**	0.29 ns	0.44 ns	0.20 ns	1.15 ns	1.00	1.14 ns	0.86 ns	1.00 ns	0.93 ns	1.00	1.16 ns	1.03 ns	1.39 *	1.11 ns	1.00
	**ITGB3**	1.67 ns	3.17 **	0.74 ns	1.57 *	1.00	2.33 **	1.88 *	0.83 ns	1.45 *	1.00	1.76 ns	1.55 ns	1.30 ns	0.70 **	1.00
	**YAP1**	0.90 *	0.52 ****	0.90 ns	0.60 *	1.00	1.31 ns	1.16 ns	1.34 ns	0.85 ns	1.00	0.87 **	0.55 ***	0.93 ns	0.41 ***	1.00
	**LMNB1**	1.18 **	0.45 ***	0.99 ns	1.04 ns	1.00	0.97 ns	1.22 ns	1.03 ns	1.51 ns	1.00	0.50 **	0.89 ns	0.63 ns	0.32 ***	1.00
	**ACTR2**	0.89 *	0.77 *	0.64 *	0.89 ns	1.00	1.42 ns	1.34 ns	1.06 ns	1.20 ns	1.00	1.31 *	0.84 ns	0.69 ns	0.83 ns	1.00
**Expression**		>10		>5		>1			=1		<1		<0.5		<0.1	
***p*-values**		ns = not significant				* = *p* < 0.05			** = *p* < 0.01		*** = *p* < 0.001			**** = *p* < 0.0001		

**Table 4:** Consequences of the individual RNAi interventions in GK, EPI and FIB cells on various protein residual activities by Western blot, presented in mean values (MV) and *p*-values.

Western Blot		FIBs					EPIs					GKs				
		KRT5-RNAi	KRT14-RNAi	IVL-RNAi	VIM-RNAi	nt-RNAi	KRT5-RNAi	KRT14-RNAi	IVL-RNAi	VIM-RNAi	nt-RNAi	KRT5-RNAi	KRT14-RNAi	IVL-RNAi	VIM-RNAi	nt-RNAi
**MV**	**KRT5**	-	-	-	-	-	0.19 ****	1.60 ns	1.16 ns	1.13 ns	1.00	0.16 ****	0.59 **	0.99 ns	0.85 ns	1.00
	**KRT14**	-	-	-	-	-	-	-	-	-	-	1.06 ns	0.14 ****	1.12 ns	0.77 *	1.00
	**VIM**	0.80 ns	0.70 *	0.73 *	0.51 ****	1.00	0.63 ***	0.63 ns	0.24 ****	0.00 ****	1.00	-	-	-	-	-
	**IVL**	1.14 ns	0.97 ns	0.22 ****	0.82 ***	1.00	0.69 **	0.56 ***	0.08 ****	0.36 ****	1.00	0.50 *	0.24 **	0.07 ****	0.63 ns	1.00
	**LOR**	1.07 ns	0.77 *	1.53 *	1.37 ns	1.00	0.64 ***	0.64 *	0.80 ns	0.92 ns	1.00	1.28 *	1.01 ns	1.32 ns	1.09 **	1.00
	**CDH1**	-	-	-	-	-	0.65 *	0.87 ns	0.74 ns	0.44 **	1.00	0.71 *	0.72 *	1.32 **	1.05 ns	1.00
	**ITGB1**	1.26 ns	1.24 ns	1.18 ns	1.09 *	1.00	3.71 ns	2.33 ns	2.89 *	1.61 *	1.00	1.74 ns	0.62 ns	1.89 ns	2.80 ns	1.00
	**ITGB3**	-	-	-	-	-	-	-	-	-	-	-	-	-	-	-
	**YAP**	1.04 ns	0.81 *	1.04 ns	1.10 ns	1.00	1.20 ns	1.28 ns	1.29 ns	1.15 ns	1.00	0.63 *	0.93 ns	1.16 ns	1.04 ns	1.00
	**LMNB1**	0.90 **	0.88 ns	1.33 *	0.92 ns	1.00	0.77 *	1.00 ns	0.78 ns	0.77 ***	1.00	0.56 **	1.10 ns	1.07 ns	0.79 *	1.00
	**ARP2/3**	-	-	-	-	-	-	-	-	-	-	-	-	-	-	-
**Expression**		>2		>1.5		>1			=1		<1		<0.5		<0.1	
***p*-values**		ns = not significant				* = *p* < 0.05			** = *p* < 0.01		*** = *p* < 0.001			**** = *p* < 0.0001		

**Table 5:** Consequences of the individual RNAi interventions on proliferation using Alamar Blue presented in mean values (MV) and *p*-values.

Proliferation		FIBs					EPIs					GKs				
		KRT5-RNAi	KRT14-RNAi	IVL-RNAi	VIM-RNAi	nt-RNAi	KRT5-RNAi	KRT14-RNAi	IVL-RNAi	VIM-RNAi	nt-RNAi	KRT5-RNAi	KRT14-RNAi	IVL-RNAi	Vim-RNAi	nt-RNAi
**Alamar Blue**	**MV**	0.84 **	0.91 ns	0.97 ns	0.99 ns	1.00	0.95 ns	0.95 ns	0.91 *	0.94 **	1.00	0.91 *	0.87 **	0.97 **	1.01 **	1.00
**Proliferation**		>1.2		>1.1		>1		=1			<1		<0.9		<0.8	
***p*** **-values**		ns = not significant				* = *p* < 0.05		** = *p* < 0.01			*** = *p* < 0.001			**** = *p* < 0.0001		

**Table 6:** Consequences of selectively evaluated RNAi interventions on the expressed amount of Eu- (H3K4me3, H3K9ac) and heterochromatin (H3K9me3, H3K27me3).

Histone State Quantification		FIBs		GKs		
		Vim-RNAi	nt-RNAi	KRT5-RNAi	KRT14-RNAi	nt-RNAi
**MV**	**H3K4me3**	0.70 ns	1.00	0.95 ns	1.07 ns	1.00
	**H3K9ac**	0.69 *	1.00	0.88 *	1.00 ns	1.00
	**H3K9me3**	0.84 ns	1.00	1.05 ns	1.09 ns	1.00
	**H3K27me3**	0.75 ns	1.00	1.01 ns	0.96 ns	1.00
Expression		>1	=1		<1	
*p*-value		ns = not significant		* = *p* < 0.05		

## Data Availability

The data presented in this study are available on request from the corresponding author.

## Supplementary Materials

The following supporting information can be downloaded at: https://www.mdpi.com/article/10.3390/cells13231960/s1, Figure S1: Indirect immunofluorescence of caspase 3 in siRNA treated gingival keratinocytes (GKs); Figure S2: Involucrin protein expression (A) and Involucrin mRNA expression in FIB, EPI GK cells, referenced to GKs. Quantification of western blot data (C) and PCR (D); Figure S3: Effectiveness of RNAi intervention at the protein level for IVL (A) and VIM (B); Figure S4: Relative mRNA expression of EMT-associated biomarkers SNAIL1 (A), ZEB1 (B) and TWIST1 (C) analysed in GKs, EPIs and FIB cells.

## Abbreviations

ALDH	alcohol dehydrogenase
ACTR2	actin-related protein 2
AD	atopic dermatitis
cIFs	cytoplasmic intermediate filaments
CE	cornified envelope
CDH1	E-cadherin
ECM	extracellular matrix
EPIs	epitheloid (epithelium-like) phenotypes
FAs	focal adhesions
FIBs	fibroblastoid (fibroblast-like) phenotypes
GKs	Gingival Keratinocytes
GGA3	Golgi-localized gamma-ear containing Arf-binding protein 3
ITGB1	intergrin β1
ITGB3	integrin β3
IVL	Involucrin
KRT1	Cytokeratin 1
KRT10	Cytokeratin 10
KRT5	Cytokeratin 5
KRT14	Cytokeratin 14
LOR	loricrin
LMNB1	lamin B1
nIFs	nuclear intermediate filaments
OSCC	oral squamous cell carcinoma
SCC	squamous cell carcinoma
SRF	serum response factor
TEAD	TEA Domain Transcription Factor1-4
TAZ	transcriptional coactivator with PDZ-binding motif
VIM	Vimentin
VCL	vinculin
YAP	yes-associated protein
