# Supplementary material for: Impairment of Intermediate Filament Expression Reveals Impact on Cell Functions Independent from Keratinocyte Transformation
Source: Cells. 2024 Nov 26;13(23):1960. doi: 10.3390/cells13231960 (PMC11640723; doi:10.3390/cells13231960)

Supplemental Figure 1:
Indirect immunofluorescence of caspase 3 in siRNA treated gingival keratinocytes (GKs). A-C siRNA-based cytokeratin 5 (KRT5) knockdown. D-E siRNA-based cytokeratin 14 (KRT14) knockdown. G-H non-targeting control (nt). Green= KRT5/KRT14, red= actin phalloidin staining, blue DAPI nuclear staining. Bars corresponds to 20x magnification in A,B,D,E,G,F and to 60x magnification in C,F,I.

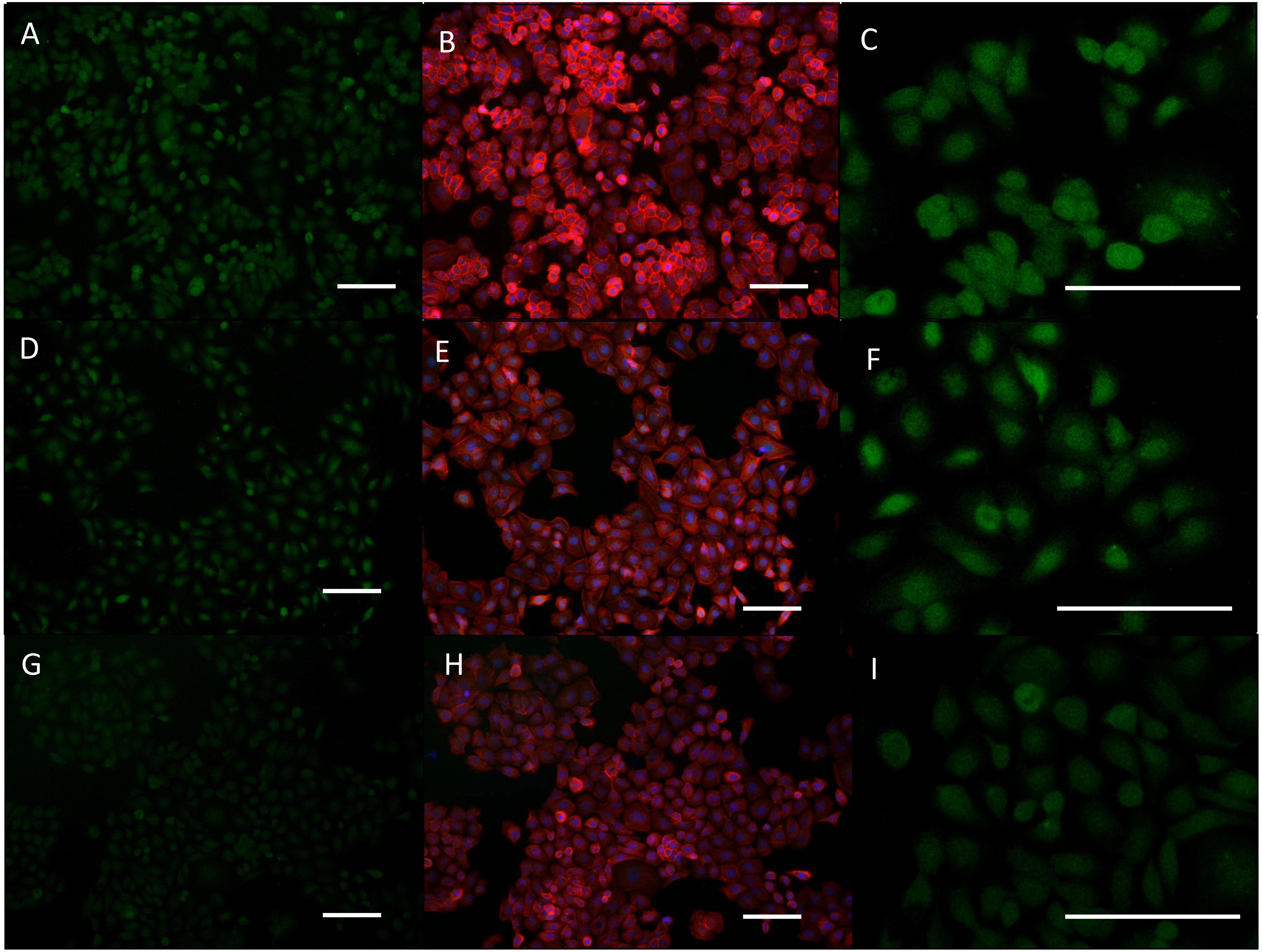

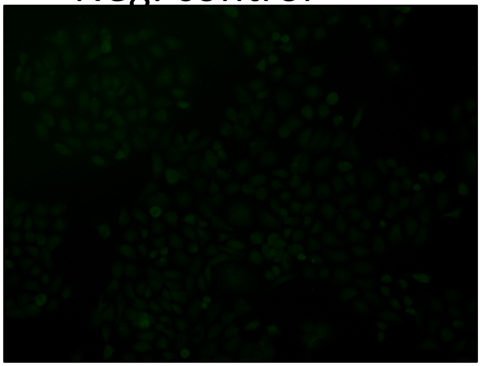

Supplement: Supplementary file 1 [file cells-13-01960-s001.zip › Supplemental Figure 1.pdf]
