# Supplementary material for: Impairment of Intermediate Filament Expression Reveals Impact on Cell Functions Independent from Keratinocyte Transformation
Source: Cells. 2024 Nov 26;13(23):1960. doi: 10.3390/cells13231960 (PMC11640723; doi:10.3390/cells13231960)
Supplement: Supplementary file 1 [file cells-13-01960-s001.zip › Supplemental Figure 2.pdf]

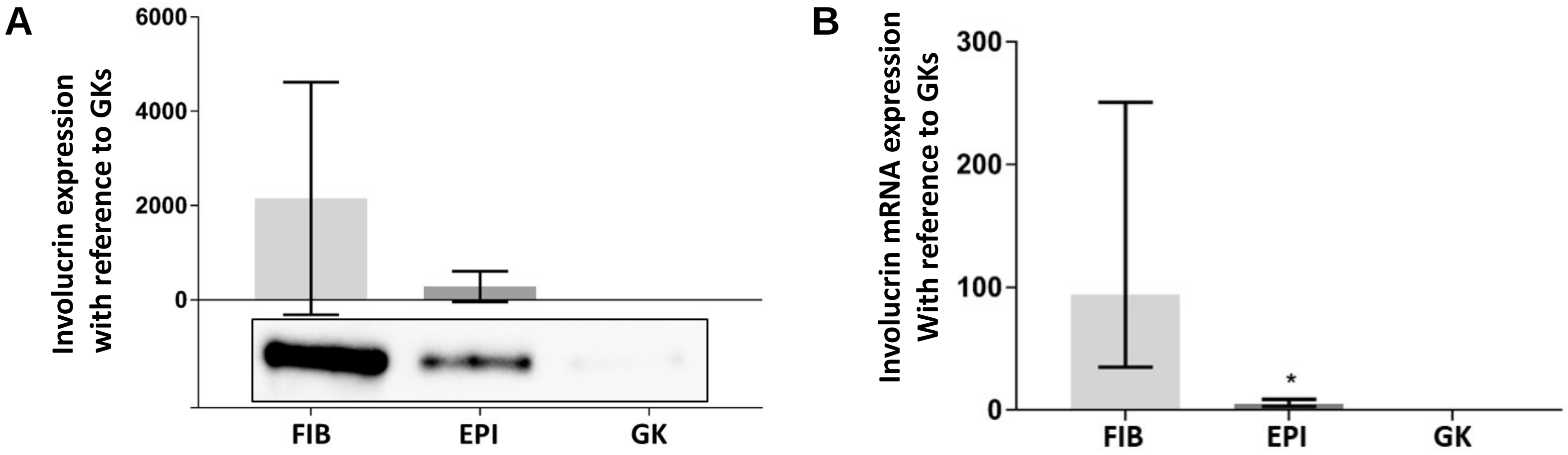

C

	FIB	EPI	GK
protein expression	2154.02	282.21	1
p-values	0.1315	0.1353	

D

	FIB	EPI	GK
mRNA expression	$2^{--6,55}$	$2^{--2,37}$	1
p-values	0.1484	0.0181	

Supplemental Figure 2:
Involucrin protein expression (A) and Involucrin mRNA expression in FIB, EPI GK cells, referenced to GKs. Quantification of western blot data (C) and PCR (D). N=3
