# Supplementary material for: Impairment of Intermediate Filament Expression Reveals Impact on Cell Functions Independent from Keratinocyte Transformation
Source: Cells. 2024 Nov 26;13(23):1960. doi: 10.3390/cells13231960 (PMC11640723; doi:10.3390/cells13231960)
Supplement: Supplementary file 1 [file cells-13-01960-s001.zip › Supplemental Figure 3.pdf]

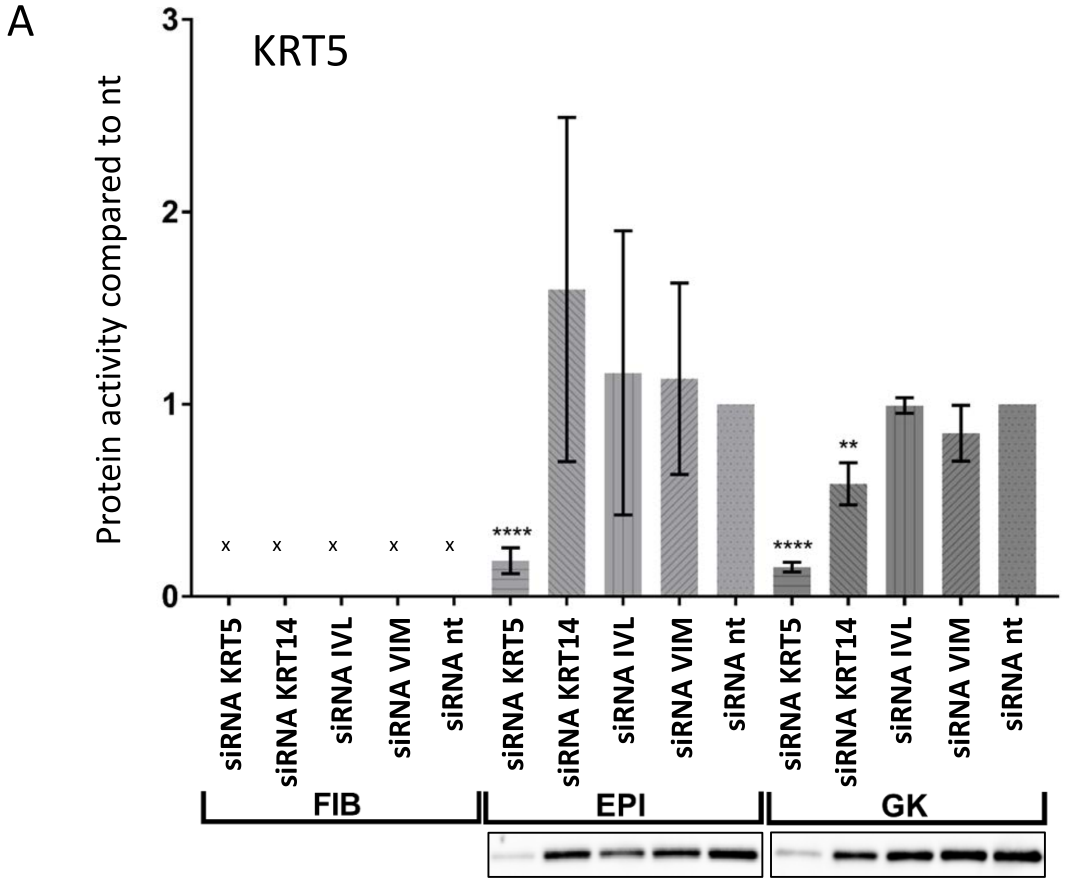


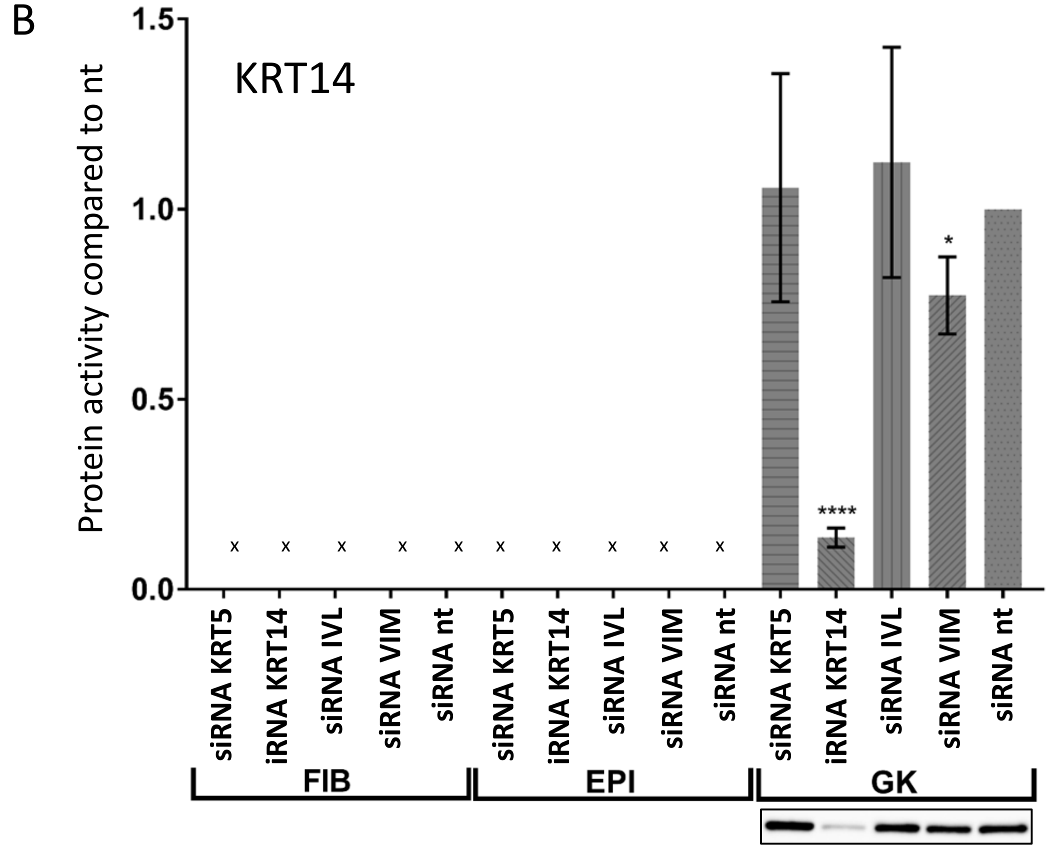


Supplemental Figure 3. Effectiveness of RNAi intervention at the protein level for KRT5 (A) and KRT14 (B). Detected is the residual activity compared to the non-targeting control and the significance levels are indicated as follows: x = not detectable; *** = p-value > 0.001; *****=p-values of >0.0001.

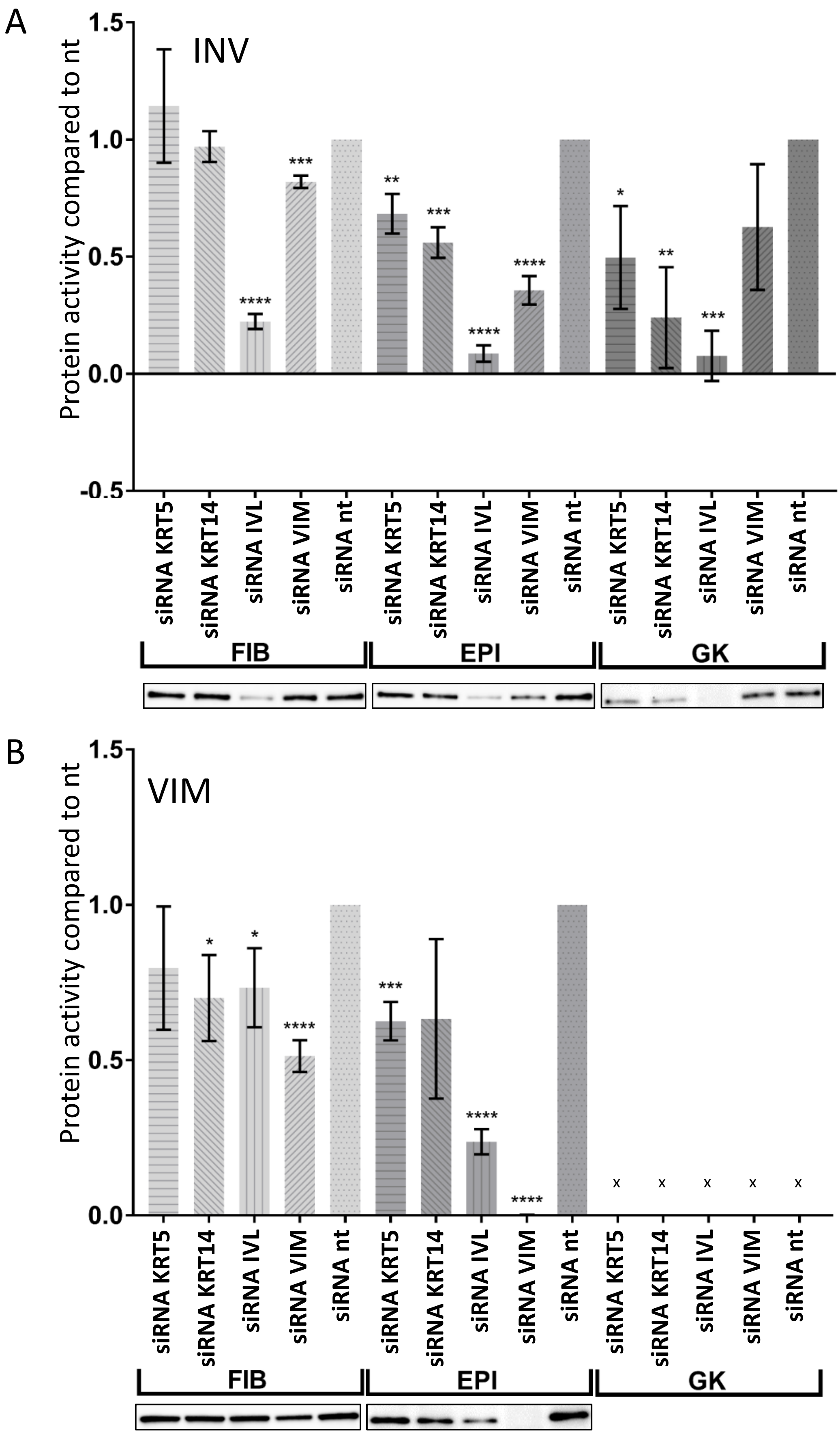


Supplemental Figure 3. Effectiveness of RNAi intervention at the protein level for INV (A) and VIM (B). Detected is the residual activity compared to the non-targeting control and the significance levels are indicated as follows: x = not detectable; *** = p-value > 0.001; *****=p-values of >0.0001.
