# Supplementary material for: Impairment of Intermediate Filament Expression Reveals Impact on Cell Functions Independent from Keratinocyte Transformation
Source: Cells. 2024 Nov 26;13(23):1960. doi: 10.3390/cells13231960 (PMC11640723; doi:10.3390/cells13231960)
Supplement: Supplementary file 1 [file cells-13-01960-s001.zip › Supplemental Figure 4.pdf]

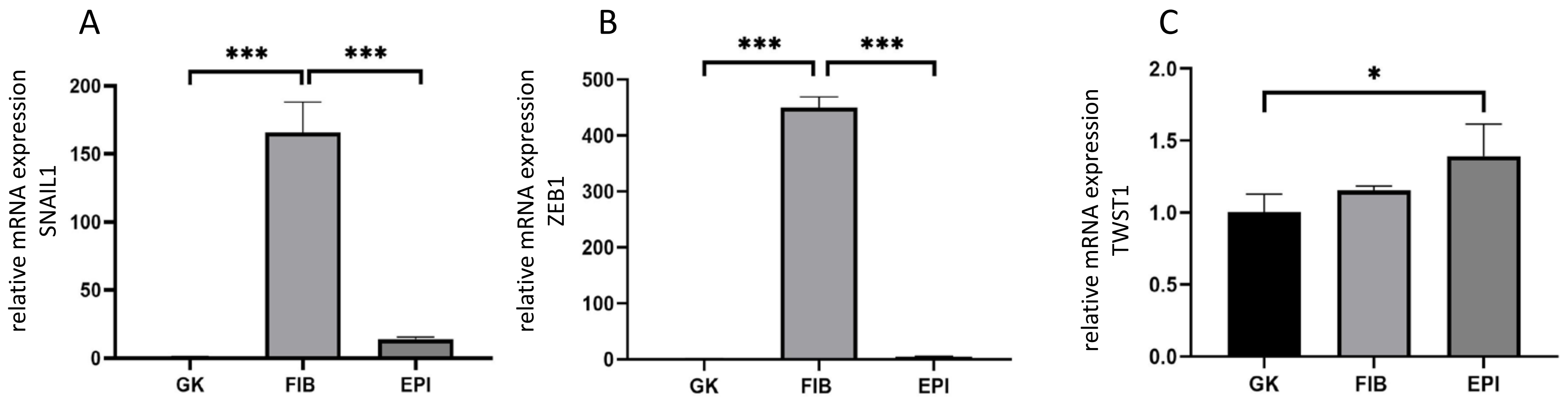

Supplemental Figur 4: Relative mRNA expression of EMT-associated biomarkers SNAIL1 (A), ZEB1 (B) and TWIST1 (C) analysed in GKs, EPIs and FIB cells. N=3,
